# An efficient multi-gram access in a two-step synthesis to soluble, nine-atomic, silylated silicon clusters

**DOI:** 10.1038/s41467-024-55211-z

**Published:** 2024-12-23

**Authors:** Kevin M. Frankiewicz, Nicole S. Willeit, Viktor Hlukhyy, Thomas F. Fässler

**Affiliations:** 1 Department of Chemistry, TUM School of Natural Sciences, Technical University of Munich (TUM), Lichtenbergstraße 4, D-85748 Garching, Germany; 2 Wacker Institute of Silicon Chemistry, Technical University of Munich (TUM), Lichtenbergstraße 4, D-85748 Garching, Germany

**Keywords:** Synthesis and processing, Organic-inorganic nanostructures, Synthetic chemistry methodology

## Abstract

Silicon is by far the most important semiconducting material. However, solution-based synthetic approaches for unsaturated silicon-rich molecules require less efficient multi-step syntheses. We report on a straightforward access to soluble, polyhedral Si_9_ clusters from the binary phase K_12_Si_17_, which contains both [Si_4_]^4−^ and [Si_9_]^4−^ clusters. [Si_4_]^4−^ ions, characterised by a high charge per atom ratio, behave as strong reducing agents, preventing [Si_9_]^4−^ from directed reactions. By the here reported separation of [Si_4_]^4−^ by means of fractional crystallisation, Si_9_ clusters of the precursor phase K_12_Si_17_ are isolated as monoprotonated [Si_9_H]^3−^ ions on a multi-gram scale and further crystallised as their 2.2.2-Cryptate salt. 20 grams of the product can be obtained through this two-step procedure - a new starting point for silicon *Zintl* chemistry, such as the isolation and structural characterisation of a trisilylated [^Me^Hyp_3_Si_9_]^−^ cluster.

## Abstract (web summary)

While silicon is a crucial semiconducting material, solution-based synthetic approaches for unsaturated silicon-rich molecules require less efficient multi-step syntheses. Here, the authors report access to soluble, polyhedral Si_9_ clusters from the binary phase K_12_Si_17_, which contains both [Si_4_]^4−^ and [Si_9_]^4−^ clusters.

## Introduction

With the progressing technologisation of our society and the accompanying miniaturisation of electronic devices, physicists and chemists face new challenges. Silicon stands out as the most important semiconducting material by far. However, traditional manufacturing methods, such as lithography and etching of crystalline silicon (top-down) or Chemical Vapour Deposition (CVD) of volatile silanes for producing nanostructured components, are reaching their limits. Nevertheless, not only manufacturing these materials requires different approaches. Bulk materials and semiconducting materials in the nanometre range significantly differ in their optical and electronic properties (quantum confinement). Molecular precursors could provide an answer to new manufacturing methods and the necessity for targeted investigations of quantum confinement effects in low-dimensional materials (quantum dots, wires and wells). Alongside the targeted synthesis of silicon nanoparticles, defined molecular (silicon) clusters are also considered model systems for studying physical and chemical processes in nanomaterials.

**Fig. 1:**
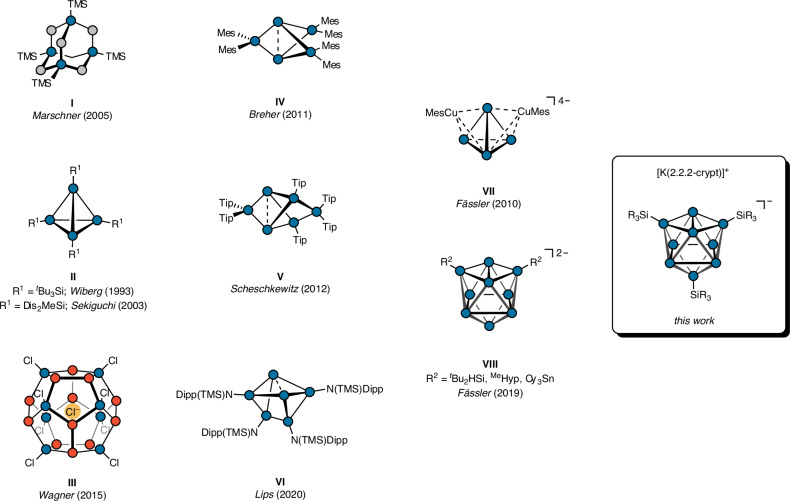
Schematic representation of selected examples of molecular silicon clusters. Examples **I**–**III** represent saturated, **IV**–**VI** siliconoid and **VII** and **VIII** *Zintl*-type clusters. Silicon is depicted as blue, SiMe_2_ units as grey and Si-SiCl_3_ units as red circles. TMS Trimethylsilyl, Dis TMS_2_HC, Mes 2,4,6-Trimethylphenyl, Tip 2,4,6-Tri-*iso*-propylphenyl, Dipp 2,6-Di-*iso*-propylphenyl, ^Me^Hyp TMS_3_Si, Cy Cyclohexyl.

The targeted synthesis of saturated cage oligosilanes^1,2^, unsaturated siliconoids^3–5^ and *Zintl* clusters^6–8^ has been intensively studied in the past decades. In 1970, West et al. achieved the preparation of a cage oligosilane under reductive conditions starting from chlorosilane precursors for the first time^9^. Such *Wurtz*-type couplings or metathesis reactions also provide access to paradigmatic clusters like silaprismanes^10–12^, -cubanes^13–16^, and -tetrahedranes^17,18^ (Fig. 1; II), primarily impressive by their structural beauty. Following West’s initial hints towards the synthesis of a permethylated sila-adamantane^19^, Marschner et al. established the synthetic pathway to the sila-adamantane derivative I (Fig. 1), representing a molecular fragment of the diamond structure of elemental silicon. *Via* a simple and elegant cascade of silyl abstraction and silylation steps, coupled with subsequent *Lewis* acid mediated isomerisation, this molecule was obtained in a stepwise synthesis from readily available TMS_4_Si^20^. Later, targeted functionalised sila-diamondoid derivatives were reported as well^21^. Apart from the synthesis of perchlorocyclohexasilane, the disproportionation of the versatile precursor Si_2_Cl_6_ also enables the synthesis of an endohedral, chloride-decorated silafullerane (Fig. 1; III)^22–24^. In addition to the described saturated silicon clusters, Breher, Scheschkewitz, and Lips report on unsaturated so-called siliconoid clusters, a term introduced by Scheschkewitz^11^. Silapropellane **IV** (Fig. 1) is obtained *via* the co-reduction of Mes_2_SiCl_2_ and Si_2_Cl_6_, exhibiting a biradicaloid character of the transannular interaction between both bridgeheads^25^. The structurally related, bridged silapropellane **V** (Fig. 1)^26^ can be derived from an aromatic dismutational isomer of hexasilabenzene in a thermal or photochemical rearrangement and serves as a starting point for a rich cluster functionalisation and expansion chemistry^27–30^. Furthermore, the suitability of amido ligands^31,32^ has been demonstrated in stabilising six-atomic silicon clusters (Fig. 1; VI), accessible *via* both the reductive coupling of corresponding bromosilane precursors and the thermal transformation of a zwitterionic tetrasilane^33^.

While all discussed routes require multi-step syntheses to build up molecules with multiple Si–Si bonds, an alternative method involves the direct formation of bare silicon clusters in a one-step synthesis by the solid-state reaction of silicon with alkali metals. This approach has yielded considerable success in the case of heavier Ge_9_ clusters. For instance, the solid-state phase M_4_Ge_9_ (M = Na–Cs)^34^ can easily be obtained and readily dissolves in various polar solvents. In recent years, a rich chemistry has been established around the nine-atomic [Ge_9_]^4−^ cluster anion such as (organo)functionalisation^35–50^, metalation^43,51–58^, and cluster growth^59–65^. In contrast to the heavier tetrel elements (Ge-Pb), [Si_9_]^4−^ cannot be selectively obtained in a solid-state reaction. Instead, only phases with the composition M_12_Si_17_ (equivalent to {(M^+^)_12_([Si_4_]^4−^)_2_([Si_9_]^4−^); M = Na–Cs) are accessible, which also contain four-atomic [Si_4_]^4−^ clusters in addition to the desired nine-atomic clusters^66^. Due to the highly reductive character of these tetrahedral ions, direct reactions of K_12_Si_17_ with electrophilic reagents like chlorosilanes are not feasible. As a result, silicon-based *Zintl* cluster chemistry has lagged behind the heavier homologues up to now and is limited to reactions and studies in liquid ammonia. Apart from bare anions [Si_9_]^x−^ (x = 2–4)^67–69^ and protonated ions like [Si_4_H]^3−^^70^ and [Si_9_H_n_]^(4-n)−^ (n = 1, 2)^71–73^ there are only six examples of metalated silicon-based *Zintl* ions, such as [PhZnSi_9_]^3−^^74^, [{Ni(CO)_2_}_2_(*μ*-Si_9_)_2_]^8−^^75^, [NHC^Dipp^Cu(*η*^4^-Si_9_)H]^2−^^76^, and [(NHC^*t*Bu^Au)_6_(*η*^2^-Si_4_)]^2−^^77^, as well as [(MesCu)_2_Si_4_]^4−^ (Fig. 1; VII)^78^ and [NHC^Dipp^Cu(*η*^4^-Si_9_)]^3−^^79^. Both the removal of the highly reactive four-atomic silicon clusters and the avoidance of liquid ammonia as a reaction medium are crucial for further developing this kind of chemistry and overcoming synthetic limitations.

In a first step, we recently made the nine-atomic [Si_9_]^4−^ clusters available for reactions in organic solvents by dissolving K_12_Si_17_ in ammonia with 2.2.2-cryptand and subsequent solvent removal. Starting from this so-called activated precursor phase, the extraction of bis-protonated [Si_9_H_2_]^2−^ clusters in pyridine and the transformation into disubstituted dianions of the form [^2^R_2_Si_9_]^2−^ (Fig. 1; XIII; ^2^R = ^Me^Hyp, ^*t*^Bu_2_HSi, Cy_3_Sn) in thf was achieved^72,80,81^. However, the four-atomic clusters continue interfering with the respective electrophilic reagents, causing limited functional group tolerance, poor product purity and low yields.

In this work, we report on wet chemical access to a synthetic K_4_Si_9_ analogue *via* separation of four- and nine-atomic clusters in liquid ammonia through fractional crystallisation. Obtaining such a precursor compound represents a key step in the still largely unexplored chemistry of nine-atomic silicon clusters and allows for the selective synthesis of trisilylated [K(2.2.2-crypt)][(R_3_Si)_3_Si_9_] cluster salts.

## Results

### Separation of [Si_4_]^4−^ and [Si_9_]^4−^ in liquid ammonia

Our previous work shows that the solid-state phase K_12_Si_17_ can be converted into an activated form, accessible for follow-up reactions like silylation. This conversion is done by dissolving K_12_Si_17_ in liquid ammonia with 2.2.2-cryptand as a sequestering agent and subsequent solvent removal^80,81^. Nevertheless, interfering four-atomic clusters might still be present in this activated phase.

**Fig. 2:**
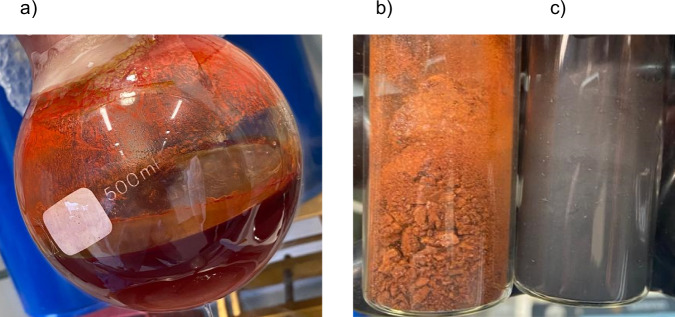
Images of cluster separation. **a** Ammonia solution of K_12_Si_17_ and 2.2.2-cryptand after storage at −40 °C for 12 h; **b** K_1-x_[K(2.2.2-crypt)]_2+x_[Si_9_] (x = 0.2) obtained from the filtrate after solvent removal; **c** Highly reactive filtration residue after solvent removal.

**Fig. 3:**
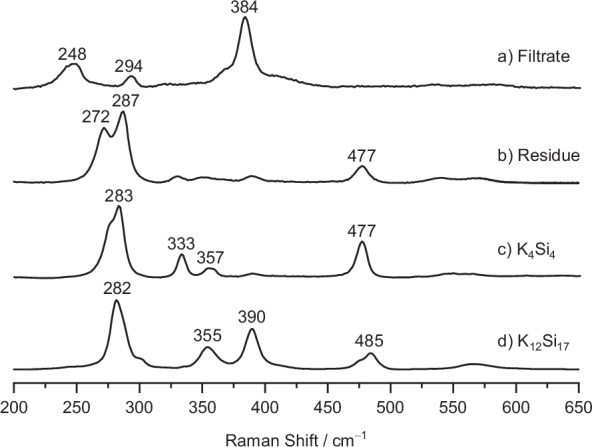
Comparative Raman analysis. **a** Filtrate, **b** filtration residue, **c** K_4_Si_4_, and **d** K_12_Si_17_.

In order to separate [Si_4_]^4−^ and [Si_9_]^4−^, we exploit their different solubilities in liquid ammonia. Keeping the ammonia extract of K_12_Si_17_ for ~12 h at −40 °C, we observe the formation of a bright red solid under a reddish-brown solution (Fig. 2a). After filtration and solvent removal, an orange, coarse solid (Fig. 2b) was isolated from the filtrate. In contrast, the former red filtration residue changed into a grey, finepowder (Fig. 2c). Surprisingly, the Raman measurements of the filtrate and filtration residue after solvent removal (Fig. 3a, b) show a clear separation of the four- and nine-atomic cluster species. For the residue, the most intense resonances at 477 cm^−1^, 287 cm^−1^, and 272 cm^−1^ can be assigned by comparison with the solid-state phase K_4_Si_4_ (Fig. 3c), which contains exclusively four-atomic [Si_4_]^4−^ clusters. The slight shift in the resonances is due to the non-identical chemical environment within the crystalline solid and the amorphous filtration residue. In contrast, the Raman spectrum of the filtrate does not show any Si_4_ band, as can be detected in K_4_Si_4_ and K_12_Si_17_ (Fig. 3d). The resonances at 294 cm^−1^ and 384 cm^−1^ agree with the Raman data already described by *Schnering* for Cs_4_Si_9_, which was obtained by the thermal decomposition of Cs_4_Si_4_^82^. The third resonance at 248 cm^−1^ indicates a further mode due to the different symmetry with respect to the *C*_*4v*_ symmetric [Si_9_]^4−^ ion.

Although we can clearly demonstrate that four- and nine-atomic clusters are separated by this procedure, the exact chemical composition of the dried filtrate cannot be conclusively determined with respect to the number of sequestered cations. We chose the minimum amount of expensive 2.2.2-cryptand, and elemental analysis of the solid shows a composition corresponding to K_1-*x*_[K(2.2.2-crypt)]_2+*x*_[Si_9_] (*x* = 0.2). The exact 2.2.2-cryptand content in this intermediate after filtration may vary around an ideal composition of K_1_[K(2.2.2-crypt)]_2_[Si_9_], which, however, had no impact on the follow-up chemistry.

**Fig. 4:**
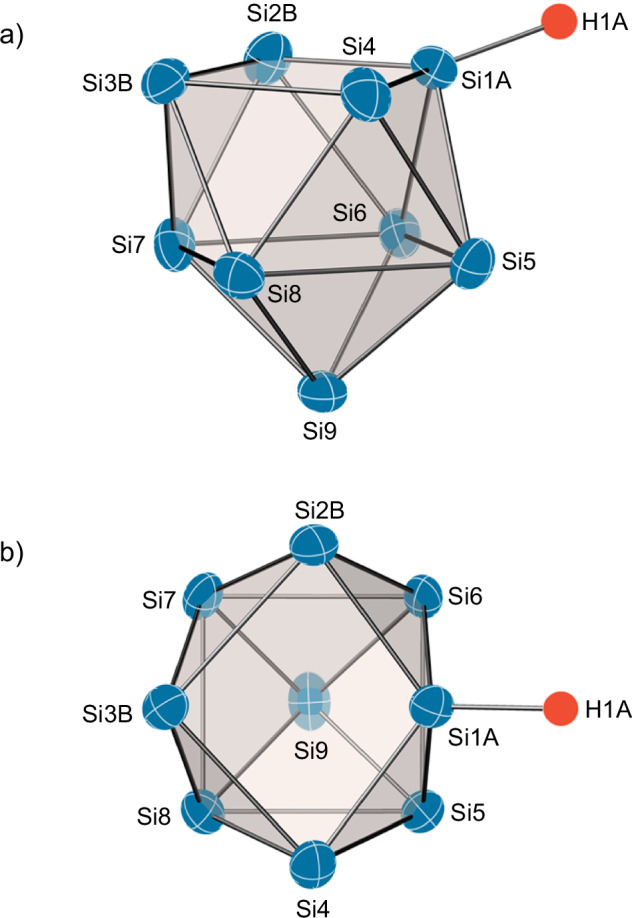
Molecular structure of the anionic cluster moiety [Si_9_H]^3-^ (α-orientation) in [K(2.2.2-crypt)]_3_[Si_9_H]·8.5NH_3_ (1). **a** Front view; **b** top view. Anisotropic displacement ellipsoids of silicon (blue) are drawn at 50% probability. The hydrogen atom (red) is displayed as a sphere of an arbitrary radius. Silicon and hydrogen atoms of minor disorder components are omitted for clarity. Selected bond length (Å): Si1A-H1A: 1.55(3); Si1A-Si2B: 2.347(3); Si1A-Si3B: 3.121(4); Si1A-Si4: 2.322(2); Si1A-Si5: 2.4244(10); Si1A-Si6: 2.4342(9); Si2B-Si3B: 2.580(2); Si2B-Si4: 3.755(3); Si2B-Si6: 2.5193(12); Si2B-Si7: 2.4655(12); Si3B-Si4: 2.532(2); Si3B-Si7: 2.4553(9); Si3B-Si8: 2.4325(9); Si4-Si5: 2.4905(7); Si4-Si8: 2.4268(7); Si5-Si9: 2.4474(8); Si6-Si9: 2.4326(8); Si7-Si9: 2.4348(8); Si8-Si9: 2.4539(7); Si2B-Si4/Si1A-Si3B: 1.20.

Single crystals as orange blocks suitable for SC-XRD were obtained by vapour diffusion of Et_2_O into an ammonia solution of the filtrate by adding additional 2.2.2-cryptand to sequester all cations. The structure determination results in the composition of [K(2.2.2-crypt)]_3_[Si_9_H]·8.5 NH_3_ (**1**). The crystal structure analysis unambiguously shows the formation of a threefold negatively charged cluster. The exceptionally good data quality allows for further structure refinement and the localisation of a hydrogen atom at the cluster from the difference Fourier map and a free refinement of the position of the H atom. The asymmetric unit contains a monoprotonated, threefold negatively charged [Si_9_H]^3−^ cluster, three [K(2.2.2-crypt)]^+^ counter ions and 8.5 equivalents of co-crystallised ammonia. The co-crystallised ammonia primarily occupies the voids between the [K(2.2.2-crypt)]^+^ units and the cluster. This results in a particular thermal and mechanical sensitivity of the crystals. The refinement shows the presence of an orientationally disordered *C*_*s*_ symmetric [Si_9_H]^3−^ cluster (for details, see the Supplementary Discussion). For the main orientation α (Fig. 4), the refinement allows for the localisation of the proton H1A at the Si1A position with a bond length of 1.55(3) Å, which is in the range of typical Si–H bond distances. The cluster framework shows the expected involvement of the substituted Si1A position of the open square plane. Thus, the Si1A-Si2B (2.347(3) Å) and Si1A-Si4 (2.322(2) Å) distances are significantly shortened compared to the Si2B-Si3B (2.580(2) Å) and Si3B-Si4 (2.532(2) Å) distances of the open square plane. The ratio of the square diagonals (Si2B-Si4/Si1A-Si3B) of 1.20 clearly shows the deviation from the ideal *C*_*4v*_ symmetry of the parent [Si_9_]^4−^ ion^69^. The structural characteristic of shorter Si-Si bonds at cluster atoms with ligands supports the existence of an H atom at Si1A and agrees with findings for the solvate [K(DB-18-crown-6)][K(2.2.2-crypt)]_2_[Si_9_H]·NH_3_^71,73^.

**Fig. 5:**
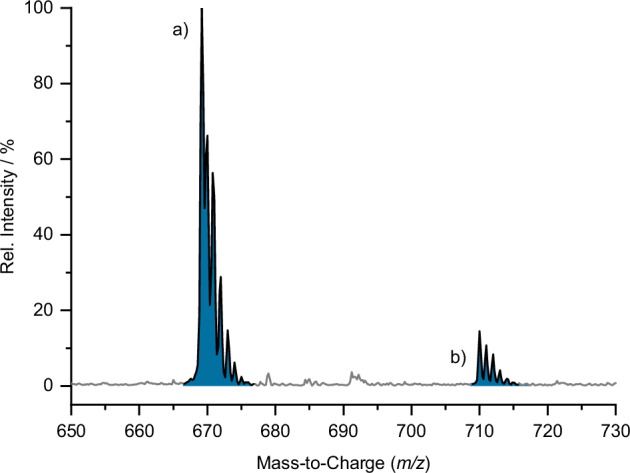
ESI(−) MS spectrum of the dried filtrate in MeCN. **a** {[K(2.2.2-crypt)][Si_9_] + 2H}^−^ (m/z = 670.37); **b** {[K(2.2.2 crypt)][Si_9_] + 2H + mecn}^−^ (m/z = 711.42).

**Fig. 6:**
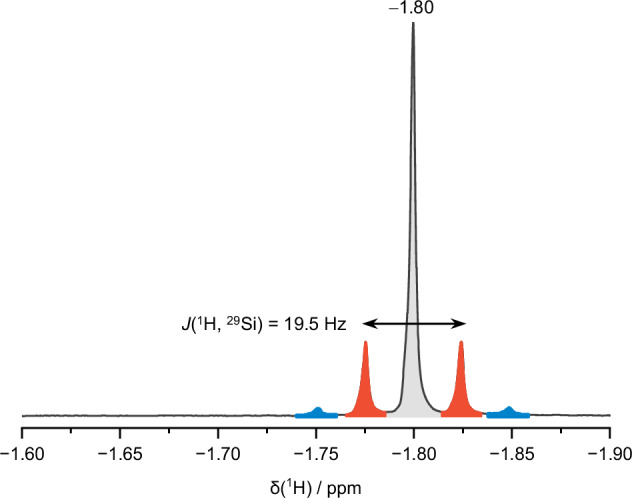
^1^H NMR spectrum of the dried filtrate. Main resonance of non-coupled isotopologues [^28/30^Si_9_^29^Si_0_H]^3−^ is indicated in grey, doublet splitting of [^28/30^Si_8_^29^Si_1_H]^3−^ in red and visible part of triplet splitting of [^28/30^Si_7_^29^Si_2_H]^3−^ in blue. (400 MHz, DMF-*d*_7_, 300 K).

The crystallographic data are supported by mass spectra of acetonitrile (MeCN) solutions of the filtration residue showing Si_9_ species (Fig. 5), confirming the presence of nine-atomic clusters in the dried filtration product. Additionally, ^1^H NMR (Fig. 6) of K_1-*x*_[K(2.2.2-crypt)]_2+*x*_[Si_9_] in DMF-*d*_7_ verifies the existence of monoprotonated cluster species. The prominent signal in the high-field region at −1.80 ppm aligns with the spectral range reported for [Si_9_H_2_]^2−^^72^. The characteristic satellite pattern emerges from scalar coupling to all nine cluster atoms. This pattern displays the superposition of all possible isotopologues caused by the low natural abundance of NMR active ^29^Si (Natural abundance = 4.7%) in the cluster framework. Consequently, the intense main singlet (indicated in grey) results from all non-NMR active isotopologues ([^28/30^Si_9_H]^3−^). While the first set of satellites (indicated in red) is due to a doublet splitting of the isotopologues [^28/30^Si_8_^29^Si_1_H]^3−^, the second set is due to a triplet splitting (indicated in blue) of the isotopologues [^28/30^Si_7_^29^Si_2_H]^3−^. The satellite signals of higher isotopologues are not detectable due to the low natural abundance of ^29^Si. A full overview of the statistical intensity distribution for the superposition of all isotopologues is given in the Supplementary Information (Supplementary Fig. 17 and Supplementary Table 7) and in accordance with previous work^72^. The exceptionally small coupling constant of *J*(^1^H, ^29^Si) = 19.5 Hz and the interaction of the proton with all nine silicon atoms of the cluster framework paints the picture of a highly dynamic system at room temperature. At −50 °C, however, a doublet with a significantly increased coupling of 152 Hz is observed (see Supplementary Fig. 18), falling within the typical range of localised ^1^*J*(^1^H, ^29^Si) couplings. Thus, proton migration becomes slow on the ^1^H NMR timescale at the transition from the high- to the low-temperature limit of proton migration, allowing for direct detection of a localised Si–H unit. This spectroscopic behaviour perfectly aligns with previously reported data in liquid ammonia^71^. Similar ligand migrations have also been described for [Sn_9_R^3^]^3−^ (R^3^ = H^83^, SnCy_3_^84^) at room temperature.

In order to clarify the origin of the cluster attached proton, we repeated the whole synthetic protocol of separation of [Si_4_]^4−^ and [Si_9_]^4−^ in ND_3_ instead of NH_3_ as solvent. After this, a signal at −1.62 ppm for [Si_9_D]^3−^ can only be detected in the ^2^H NMR (Supplementary Fig. 19). This shows that the proton of [Si_9_H]^3−^ originates from ammonia.

### Reactivity study of [Si_9_H]^3−^

**Fig. 7:**
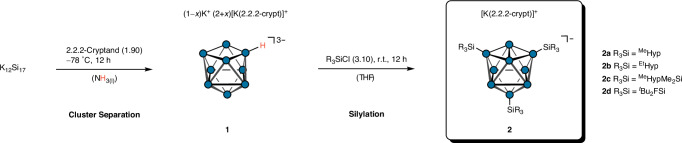
Two-step synthesis of trisilylated silicon clusters 2 *via* protonated cluster species 1. Silicon is depicted as blue circles. ^R^Hyp = (R_3_Si)_3_Si.

**Fig. 8:**
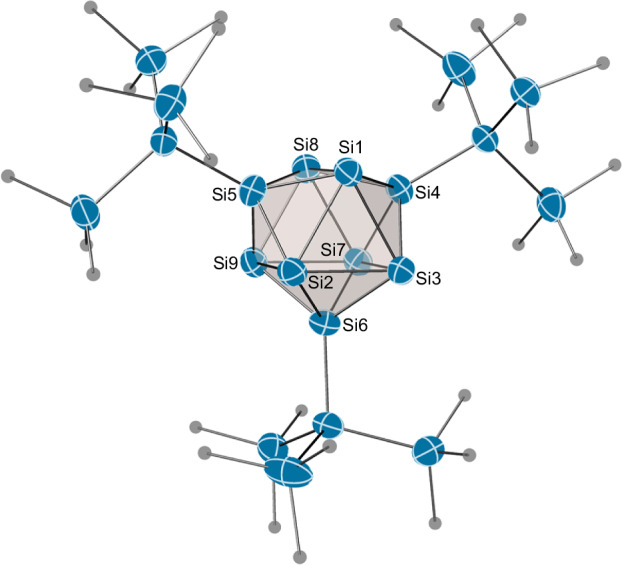
Molecular structure of the anionic cluster moiety [^Me^Hyp_3_Si_9_]^−^ in [K(2.2.2-crypt)][^Me^Hyp_3_Si_9_]·thf (2a). Anisotropic displacement ellipsoids of silicon (blue) are drawn at 50% probability. Carbon (grey) is displayed as spheres of an arbitrary radius and hydrogen atoms are omitted for clarity.

The access to isolated Si_9_ clusters on a multi-gram scale, in the absence of highly reductive [Si_4_]^4−^ clusters, provides promising and well-defined conditions for follow-up reactions of nine-atomic silicon clusters. Silylation reactions of K_1-*x*_[K(2.2.2-crypt)]_2+*x*_[Si_9_] in tetrahydrofuran (thf) lead to the isolation and the structural characterisation of trisilylated cluster salts [K(2.2.2-crypt)][(R_3_Si)_3_Si_9_] (**2a**–**2d**; Fig. 7) in good yields as orange-brown solids. All compounds were characterised by NMR and ESI-MS analyses. Yellow block-shaped single crystals of [K(2.2.2-crypt)][^Me^Hyp_3_Si_9_]·thf were grown from a thf solution at −32 °C over two weeks. The crystal structure of the molecular anion in **2a** is depicted in Fig. 8. **2a** crystallises in the monoclinic space group *P2*_*1*_*/n* (14) (a = 15.0913(4) Å, b = 24.7859(6) Å, c = 23.9571(6) Å, α = 90°, β = 90.959(2)°, γ = 90°, V = 8959.9(4) Å^3^) with one trisilylated [^Me^Hyp_3_Si_9_]^−^ cluster anion, one disordered [K(2.2.2-crypt)]^+^ unit and one disordered thf molecule in the asymmetric unit (for more details see Supplementary Discussion). Analogously to the homologous germanium cluster^85^, the present silicon cluster can also be described as a *D*_*3h*_ symmetric threefold capped trigonal prism. The attachment of a further hypersilyl group to the *C*_*2v*_ symmetric dianion [^Me^Hyp_2_Si_9_]^2−^^81^ leads to a closure of the planar square plane Si1-Si4-Si8-Si5 by shortening of the Si1-Si8 bond from 3.770(7) Å to 3.2565(10) Å. At the same time, the remaining prism edges (Si2-Si9 and Si3-Si7) are elongated by 0.526 Å and 0.430 Å, respectively. The attachment of the third silyl substituent at the Si_9_ cluster induces the same geometric changes that have been described for the homologous [^Me^Hyp_n_Ge_9_]^(4-n)−^ (Supplementary Table 6)^41,85^ and [^Me^Hyp_n_Sn_9_]^(4-n)−^ clusters (n = 2, 3)^86,87^. As expected, the cluster framework undergoes a significant contraction from tin and germanium to silicon. Similar to the homologous germanium cluster [^Me^Hyp_3_Ge_9_]^−^^47^, the UV-VIS spectra of **2a** and **2d** in thf (Supplementary Figs. 33 and 34) exhibit intense, overlapping signals below 400 nm. The attachment of the electron-withdrawing silyl ligand ^*t*^Bu_2_FSi in **2d** results in a hypsochromic shift compared to **2a**.

As expected from the crystal structure and analogously to [^Me^Hyp_3_Ge_9_]^−^^85^, **2a** also behaves *D*_*3h*_ symmetric on NMR time scale in thf-*d*_8_ at room temperature. Hence, the three hypersilyl groups collapse to two ^29^Si resonances at −130.03 ppm and −8.71 ppm. These signals can be attributed to the *exo*-bonded silicon atoms (*Si*TMS_3_) and the TMS groups, respectively. Further, in the high field region at −175.3 ppm, the three capping positions show one signal, while the six equivalent prism positions exhibit a strongly shielded signal at −360.8 ppm. The NMR data are consistent with the data described in our previous studies^81^. The remaining derivatives **2b**–**d** exhibit similar behaviour, with the cap and prism signals falling within the characteristic range of −160 ppm and −350 ppm. The high quality of the data enables the determination of the ^1^*J*(^29^Si, ^29^Si) homonuclear couplings between the cap and prism atoms (Table 1). The coupling constants of the sterically demanding hypersilyl groups (40.1 Hz for **2a** and 42.7 Hz for **2b**) are significantly higher than those of the sterically less demanding silyl groups in **2c** (24.4 Hz) and **2d** (23.2 Hz). These couplings differ from localised Si–Si bonds as in cyclic oligosilanes (^1^*J*(^29^Si, ^29^Si) ≈ 50–70 Hz)^88^ and may indicate possible dynamic processes in the cluster framework, which have not yet been described in homologous silylated clusters. The access to NMR active cluster frameworks could reveal processes that have remained hidden from us so far.

**Table 1:** Selected spectroscopic data of **2a**–**2d**

R_3_Si	δ(Si_cap_)	δ(Si_cap_)	^1^*J*(^29^Si_Cap_, ^29^Si_Prism_)
^Me^Hyp (**2a**)	−175.3	−360.8	40.1
^Et^Hyp (**2b**)	−171.3	−351.8	42.7
^Me^HypMe2Si (**2c**)	−146.3	−347.4	24.4
^*t*^Bu_2_FSi (**2d**)^a^	−177.7	−357.37	23.2

^a1^*J*(^19^F, ^29^Si) = 340.9 Hz; ^2^*J*(^19^F, ^29^Si) = 19.4 Hz.

^29^Si NMR shifts of silylated cluster positions (Si_Cap_) and prismatic positions (Si_prism_) and homonuclear *J* coupling (298 K, 79.5 MHz, thf-*d*_8_).

The presence of strongly reductive [Si_4_]^4−^ clusters in the solid-state phase K_12_Si_17_ limits the directed conversion of nine-atomic [Si_9_]^4−^ silicon clusters. However, in this work, we have shown that the separation of both cluster species is easily possible on a multi-gram scale in liquid ammonia and provides valuable synthetic access to [Si_9_H]^3−^ ions.

[Si_9_H]^3−^ shows a pronounced tautomerisation tendency, in which the proton rapidly migrates over the entire nine-atomic cluster framework. In addition, those monoprotonated silicon clusters in the form of the crude product K_1-*x*_[K(2.2.2-crypt)]_2+*x*_[Si_9_] represent a synthetic equivalent to [Si_9_]^4−^ ions that are still not accessible in an isolated form *via* a solid-state approach. Thus, the trisilylated cluster salts [K(2.2.2-crypt)][(R_3_Si)_3_Si_9_] (**2a**–**d**) are obtained in good yields and high purity by direct silylation of K_1-*x*_[K(2.2.2-crypt)_2+*x*_[Si_9_H] with the corresponding chlorosilanes. The spectroscopic behaviour and the crystallographic characterisation of **2a** prove the strong similarities between silicon- and germanium-based *Zintl* clusters. With the present work, we were able to close a significant gap in the chemistry of group 14 *Zintl* ions. Studies on the further reactivity of the obtained trisilylated monoanions are underway.

## Methods

### General

All reactions and manipulations were performed in oven dried glassware under a purified argon atmosphere using standard *Schlenk* and glove box techniques unless otherwise mentioned. NMR solvents were purchased from Sigma–Aldrich and stored over molecular sieve (3 Å) for at least one day. Dichloromethane, Tetrahydrofuran (THF), and pentane were dried by using a solvent purificator (*MBraun* MB-SPS) and stored over molecular sieve (3 Å). Ammonia was liquified in a dry ice/^*i*^PrOH bath and dried over sodium metal for one night prior to use. ND_3_ was prepared from D_2_O and Mg_3_N_2_. Triethylene glycol bis(*p*-toluenesulfonate) was prepared by a modified literature procedure^89^.

### Synthesis of K_12_Si_17_

A mixture of potassium (1.49 g, 38.0 mmol, 12.0 eq.) and silicon (1.51 g, 53.9 mmol, 17.0 eq.) was sealed in a tantalum ampule and heated up to 800 °C with a rate of 2 K/min. After 18 h, the reaction mixture was cooled down to room temperature (1 K/min) yielding K_12_Si_17_ (2.91 g, 97%) as a dark grey microcrystalline solid. The analytical data (Supplementary Fig. 9) agree with the literature^72^.

### Synthesis of K_4_Si_4_

A mixture of 350 mg potassium (8.95 mmol, 1.00 eq.) and 251 mg silicon (8.95 mmol, 1.00 eq.) was sealed in a tantalum ampule and heated up to 600 °C with a rate of 2 K/min. After ten hours, the reaction mixture was cooled down to room temperature (1 K/min) yielding K_4_Si_4_ (589 mg, 98%) as a dark grey microcrystalline solid. The analytical data (Supplementary Fig. 10) agree with the literature^72^.

### Synthesis of triethylene glycol bis(*p*-toluenesulfonate)

45.1 g triethylen glycol (300 mmol, 1.00 eq.) was dissolved in 300 mL CH_2_Cl_2_ under non-inert conditions. After addition of 114 g TsCl (600 mmol, 2.00 eq.), the mixture was cooled to 0 °C and 135 g powdered KOH (2.40 mol, 8.00 eq.) was carefully added in small portions (*Caution: Can cause strong heat evolution*). After stirring for three hours at 0 °C, 300 mL CH_2_Cl_2_ and 600 mL ice-water were added. The organic layer was separated and the aqueous phase was extracted with CH_2_Cl_2_ (3 × 200 mL). The combined organic layers were washed with water (2 × 100 mL), dried over Na_2_SO_4_ and rotary evaporated. Triethylene glycol bis(*p*-toluenesulfonate) (118 g, 258 mmol, 86%) was obtained as a white solid. ^**1**^**H NMR** (400 MHz, CDCl_3_, 298 K): δ 7.77 (d, *J* = 8.0 Hz, 4 H), 7.33 (d, *J* = 8.0 Hz, 4 H), 4.12 (t, *J* = 4.8 Hz, 4 H), 3.63 (t, *J* = 4.8 Hz, 4 H), 3.50 (s, 4 H), 2.42 (s, 6 H); ^**13**^**C{**^**1**^**H} NMR** (100 MHz, CDCl_3_, 298 K): δ 145.0, 133.0, 129.9, 128.0, 70.7, 69.3, 68.8, 21.7. The analytical data agree with the literature^89^.

### Synthesis of 2.2.2-cryptand

A mixture of 31.0 g triethylene glycol bis(*p*-toluenesulfonate) (67.0 mmol, 2.00 eq.), 62.1 g of Na_2_CO_3_ (586 mmol, 17.5 eq.) and 4.89 mL 2,2′-(ethylenedioxy)bis(ethylamine) (33.5 mmol, 1.00 eq.) was refluxed in 1000 mL MeCN for five days under non-inert conditions. After cooling to room temperature, the mixture was filtrated and rotary evaporated. The resulting orange oil was redissolved in 375 mL EtOH and 50.0 mL citric acid (1.8 m), heated to 85 °C for three hours and filtrated again. After adjusting the pH of the filtrate to 14 with aqueous tetramethylammonium hydroxide solution, the mixture was rotary evaporated. The resulting residue was redissolved with *Celite* in CH_2_Cl_2_ and rotary evaporated again. After *Soxhlet* extraction with cyclohexane overnight and recrystallisation from CH_2_Cl_2_:Et_2_O (1:4), 2.2.2-cryptand was obtained as a white crystalline solid (5.29 g, 14.1 mmol, 42%). Further purification was achieved *via* sublimation (0.1 mbar, 130 °C). The analytical data match with an authentic sample of 2.2.2-cryptand. ^**1**^**H NMR** (400 MHz, CDCl_3_, 298 K): δ 2.62 (t, ^3^*J* = 5.56 Hz, 12H, NC*H*_2_CH_2_), 3.56 (t, ^3^*J* = 5.56 Hz, 12H, NCH_2_C*H*_2_), 3.66 (s, 12H, CH_2_). **Elemental Analysis**: (calcd., found for C_18_H_36_N_2_O_6_) C (57.42, 57.52), H (9.64, 9.64), N (7.44, 7.44).

### Cluster separation in liquid ammonia

K_12_Si_17_ (25.0 g, 26.4 mmol, 1.00 eq) and 2.2.2-cryptand (18.9 g,50.2 mmol, 1.90 eq) were dissolved in 250 mL NH_3(l)_ at −78 °C under inert atmosphere leading to a deep red dispersion. The reaction was stirred for one hour before it was stored at −40 °C for twelve hours. After filtration under continuous cooling with dry ice, the ammonia of the deep red filtrate was evaporated. The resulting red-orange solid was weighted (20.4 g) and used without further purification for silylation experiments. According to the elemental analysis, the solid consist of K_1-*x*_[K(2.2.2-crypt)]_2+*x*_[Si_9_H] (*x* = 0.2). We would like to point out that the number of non-sequestered and sequestered potassium ions may not always have the same ratio and the exact composition of the crude product might slightly vary with respected to the number of sequestered cations. The amount of 2.2.2-cryptand was optimised in order to reach the best separation of Si_9_ and Si_4_ clusters also considering using the minimum amount of 2.2.2-cryptand to reduce the costs. We found that the follow-up chemistry of the anion [Si_9_H]^3−^ is not influenced by the amount of 2.2.2-cryptand. *Caution: The grey, dried filtration residue reacts explosively with air and protic solvents. Isolation is strongly discouraged. Even Raman measurements conducted within airtight glass capillaries have sometimes resulted in the detonation of these capillaries. Hence, it is strongly recommended to carefully quench the* ***undried residue*** *with* ^*i*^*PrOH at −78* *°C overnight! The solid passivates in* ^*i*^*PrOH. After one night, a bright red reactive solid may remain in the flask. Do not quench this solid with water or* ^*i*^*PrOH at room temperature under any circumstances! Even small amounts of this residue can react explosively*.

Red orange block-shaped single crystals (20%) of [K(2.2.2-crypt)]_3_[Si_9_H]∙8.5NH_3_ (**1**) were obtained by vapour diffusion of Et_2_O into an ammonia solution of K_1-x_[K(2.2.2-crypt)]_2+x_[Si_9_H] (1.00 eq.) and cryptand (1.00 eq.) at −40 °C after one week. ^**1**^**H NMR** (400 MHz, DMF-*d*_7_, 298 K): δ −1.80 (s, Si–*H*). **ESI-MS** (negative mode, 3500 V, 300 °C): *m/z* 670.37 ({[K(2.2.2-crypt)][Si_9_]+2H}^−^), 711.42 ({[K(2.2.2-crypt)][Si_9_]+2H+mecn}^−^); **Elemental Analysis** (calcd., found for K_1-*x*_[K(2.2.2-crypt)]_2+*x*_[Si_9_H]; *x* = 0.2; C_39.6_H_80.2_K_3_N_4.4_O_13.2_Si_9_): C (39.70, 39.66), H (6.78, 6.74), N (5.47, 5.14).

### Synthesis of ^Me^HypMe_2_SiH

1.50 g TMS_4_Si (4.68 mmol, 1.00 eq.) and 551 mg KO^*t*^Bu (4.91 mmol, 1.05 eq.) were dissolved in 7.50 mL THF and stirred for 5 h at room temperature. The resulting yellowish solution was slowly added to a solution of 442 mg Me_2_SiHCl (4.68 mmol, 1.00 eq.) in 5.00 mL THF at −78 °C. After complete addition, the reaction mixture was stirred at room temperature overnight before quenched with sat. aqueous NH_4_Cl solution. The mixture was extracted with Et_2_O (3 × 25 mL). The combined organic layers were washed with brine and dried over Na_2_SO_4_. After filtration rotary and evaporation of the solvent, ^Me^HypMe_2_SiH (1.32 g, 4.34 mmol, 93%) was obtained as colourless solid. ^**1**^**H NMR** (400 MHz, CDCl_3_, 298 K): δ 4.02 (sept, ^3^*J*(^1^H, ^1^H) = 4.4 Hz, dsept, ^1^*J*(^1^H, ^29^Si) = 177 Hz, ^3^*J*(^1^H, ^1^H) = 4.4 Hz, 1H, Si–*H*), 0.26 (d, ^3^*J*(^1^H, ^1^H) = 4.4 Hz, 6H, SiMe_2_), 0.21 (s, 27H, ^Me^Hyp); ^**13**^**C{**^**1**^**H} NMR** (101 MHz, CDCl_3_, 298 K): δ 2.63 (^Me^Hyp), −1.95 (SiMe_2_); ^**29**^**Si{**^**1**^**H} INEPT** (79.5 MHz, CDCl_3_, 298 K): δ −9.42 (TMS), −33.51 (SiMe_2_), −136.59 (TMS_3_*Si*).

### Synthesis of ^Me^HypMe_2_SiCl

1.00 g ^Me^HypMe_2_SiH (3.26 mmol, 1.00 eq.) and 277 mg TCCA (1.19 mmol, 0.37 eq.) were stirred in 2.00 mL CH_2_Cl_2_ overnight under formation of a white suspension. After solvent removal under reduced pressure, the resulting white solid was extracted with pentane (3 × 15 mL). The combined solutions were evaporated under reduced pressure, giving ^Me^HypMe_2_SiCl as colourless solid. ^**1**^**H NMR** (400 MHz, MeCN-*d*_3_, 298 K): δ 0.61 (s, 6H, SiMe_2_), 0.26 (s, 27H, ^Me^Hyp). The analytical data agree with the literature^90^.

### General procedure for the synthesis of [K(2.2.2-crypt)][(R_3_Si)_3_Si_9_] (2)

K_1-x_[K(2.2.2-crypt)]_2+x_[Si_9_H] (x = 0.2) (1.00 eq.) and chlorosilane (3.10 eq.) were dissolved in thf and stirred at room temperature under formation of a red-brown solution. After filtration and removing of the solvent *in vacuo*, the resulting solid was washed with pentane. After drying under reduced pressure, the trisilylated cluster salts [K(2.2.2-crypt)][(R_3_Si)_3_Si_9_] (**2**) were obtained as orange-brown solids.

### Single-crystal X-ray diffraction (SC-XRD)

Crystal preparation was carried out under a continuous flow of cold nitrogen in perfluorinated ether (Galden® LS 230, Solvay Specialty Polymers Italy SpA). For single-crystal data collection, the crystals were fixed on a glass capillary and positioned in a cold stream (150 K) of dried N_2_ gas. Single-crystal data collection was performed with a STOE StadiVari diffractometer (Mo *Kα* radiation; λ = 0.71072 Å) equipped with a DECTRIS PILATUS 300 K detector.

The X-Area 1.9 software package (*Stoe*) was used for data reduction and absorption correction^91^. Structures were solved by Direct Methods (SHELXS-2014) and refined by full-matrix least-squares calculations against *F*^2^ (SHELXL-2014)^92,93^. The positions of the hydrogen atoms were either refined from the difference Fourier map or calculated and refined using a riding model. Unless otherwise stated, all non-hydrogen atoms were treated with anisotropic displacement parameters. The silicon cluster in compound **1** (CCDC 2338275) shows orientational disorder over three orientations and disorder of non-coordinated ammonia molecules. In compound **2** (CCDC 2232604) the disorder of [K(2.2.2-crypt)]^+^ and thf has been refined by a split layer refinement. For more details see the Supplementary Information (section Crystallographic Data). The crystal structures have been visualised with CrystalMaker® 11.1.1^94^ and Diamond 3.2^95^.

### Powder X-ray diffraction (PXRD)

The data were collected at room temperature on a *STOE Stadi P* diffractometer (Ge(111) monochromator, Cu Kα_1_ radiation, *λ* = 1.54056 Å) with a DECTRIS MYTHEN 1 K detector in Debye–Scherrer geometry. For the measurements, the samples were sealed in glass capillaries (*Ø* = 0.3 mm). The raw data were processed with WinX-POW^96^. OriginPro 2023 (OriginLab Corporation) was used for visualisation^97^.

### Nuclear magnetic resonance spectroscopy (NMR)

^1^H, ^2^H, ^13^C, ^19^F and ^29^Si NMR spectra were recorded on a Bruker AVIII Ultrashield 400 and AVIII HD 500 Cryo. The signals of the ^1^H NMR spectra were referenced to the residual proton signal and the ^13^C-NMR spectra on the ^13^C signal of the deuterated solvent. ^2^H (δ (Me_4_Si-*d*_12_) = 0 ppm), ^19^F (δ (CFCl_3_) = 0 ppm), and ^29^Si (δ (Me_4_Si) = 0 ppm) were referenced to external standards. Chemical shift values are given in δ values in parts per million (ppm). The coupling constants *J* are given in Hz. Signal multiplicities are abbreviated as follows: s—singlet, d—doublet, t—triplet, q—quartet, sept—septet, dsept—doublet of septet, b—broad. The spectra were processed and visualised with MestReNova 15.0.0^98^ and OriginPro 2023 (OriginLab Corporation)^97^.

### Raman spectroscopy

Raman measurements were performed with a Renishaw inVia Reflex Raman System with a CCD Detector (Renishaw 266n10 detector) and a 785 nm laser of 500 mW max. power (Software WiRE 5.3 Renishaw) in sealed glass capillaries (Ø = 0.5 mm)^99^. The spectra were visualised with OriginPro 2023 (OriginLab Corporation)^97^.

### Electrospray-ionisation mass spectrometry (ESI-MS)

ESI-MS spectra were measured on an HCT instrument (Bruker Inc). The data were processed with Bruker Compass Data Analysis 4.0 SP 5. The dry gas temperature was adjusted to 573 K and the injection speed to 270 μL/s. Data visualisation of the spectra was carried out with the programs OriginPro 2023 (OriginLab Corporation)^97^.

### Ultraviolet-visible spectroscopy (UV-VIS)

UV‐VIS spectra were recorded on an Agilent Cary 60 UV‐Visible spectrophotometer (Agilent Technologies). The absorption spectra were recorded in 1 mm quartz cuvettes (Hellma Analytics) in thf at room temperature. OriginPro 2023 (OriginLab Corporation) was used for visualisation^97^.

### Elemental analysis

Elemental analyses were performed by the microanalytical laboratory at the Catalysis Research Center (CRC) of the Technical University of Munich (TUM). The elements C, H, and N were determined by a combustion analyser (EURO-EA, HEKATech).

## Supplementary information

Supplementary Information

Transparent Peer Review file

## Data Availability

All data generated or analysed during this study are available in this published article, its Supplementary Information files or from the corresponding authors on request. The X-ray crystallographic coordinates for the structures reported in this study have been deposited at the Cambridge Crystallographic Data Centre (CCDC), under the deposition numbers 2338275 (**1**) and 2232604 (**2a**). These data can be obtained free of charge from The CCDC via www.ccdc.cam.ac.uk/data_request/cif.
