# Supplementary material for: An efficient multi-gram access in a two-step synthesis to soluble, nine-atomic, silylated silicon clusters
Source: Nat Commun. 2024 Dec 23;15:10715. doi: 10.1038/s41467-024-55211-z (PMC11666712; doi:10.1038/s41467-024-55211-z)
Supplement: Supplementary file 1 — Supplementary Information [file 41467_2024_55211_MOESM1_ESM.pdf]

Supplementary Information

An efficient multi-gram access in a two-step synthesis to soluble, nine-atomic, silylated silicon clusters

Kevin M. Frankiewicz[1,2], Nicole S. Willeit[1,2], Viktor Hlukhyy[1] & Thomas F. Fässler[1,2]*

[1] *Department of Chemistry, TUM School of Natural Sciences, Technical University of Munich (TUM), Lichtenbergstraße 4, D-85748 Garching, Germany.*

[2] *Wacker Institute of Silicon Chemistry, Technical University of Munich (TUM), Lichtenbergstraße 4, D-85748 Garching, Germany.*

* *Correspondence: thomas.faessler@lrz.tum.de (Thomas F. Fässler)*

Table of contents

1. Synthetic procedures for [K(2.2.2-crypt)][$(R_3Si)_3Si_9$] (**2a-2d**) S2
2. Single-crystal XRD data S4
   2.1 [K(2.2.2-crypt)]$_3$[Si_9H]·8.5NH_3 (**1**) (CCDC 2338275) S5
   2.2 [K(2.2.2-crypt)][$^{Me}Hyp_3Si_9$]·thf (**2a**) (CCDC 2232604) S10
3. Powder X-ray data S16
4. NMR data S17
5. UV-VIS spectra S30
6. Review discussion S31
7. References S33

1. Synthetic procedures for [K(2.2.2-crypt)][$(R_3Si)_3Si_9$] (2a-2d)

[K(2.2.2-crypt)][$^{Me}Hyp_3Si_9$] (**2a**).

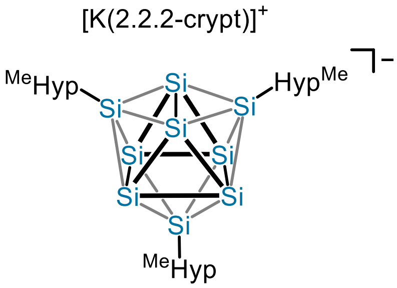

2a

$C_{45}H_{117}KN_2O_6Si_{21}$

1411.32 g/mol

K[K(2.2.2-crypt)]$_2$[Si_9H] (500 mg, 445 µmol, 1.00 eq.) and ^{Me}HypCl (390 mg, 1.38 mol, 3.10 eq.) were reacted in 10.0 mL THF. After workup, 389 mg (276 µmol, 62%) of [K(2.2.2-crypt)][$^{Me}Hyp_3Si_9$] (**2a**) were obtained as an orange-brown powder. Yellow crystals suitable for SC-XRD were grown from thf solutions at –32 °C after two weeks. **^{1}H NMR** (500 MHz, thf-d_8, 298 K): δ 0.26 (s, 81H, ^{Me}Hyp), 2.62 (t, 12H, 2.2.2-crypt), 3.60 (t, 12 H, 2.2.2-crypt), 3.65 (s, 12H, 2.2.2-crypt); **^{13}C{^{1}H} NMR** (126 MHz, thf-d_8, 298 K): δ 3.49 (s, ^{Me}Hyp), 54.93 (s, 2.2.2-crypt), 68.62 (s, 2.2.2-crypt), 71.47 (s, 2.2.2-crypt); **^{29}Si{^{1}H} IG NMR** (99.4 MHz, thf-d_8, 298 K): δ –306.77 (s, Si_{prism}), –175.33 (s, Si_{cap}), –130.03 (s, *Si*($SiMe_3$)$_3$), –8.71 (s, Si(*Si*Me_3)$_3$); **ESI-MS** (negative mode, 3500 V, 300 °C): *m/z* = 995.73 ([(^{Me}Hyp)$_3Si_9$]$^-$); **Elemental Analysis** (calcd., found for $C_{45}H_{117}KN_2O_6Si_{21}$): C (38.30, 38.40), H (8.36, 8.40), N (1.98, 2.19).

The analytical data agree with the literature.[1]

[K(2.2.2-crypt)][$^{Et}Hyp_3Si_9$] (**2b**).

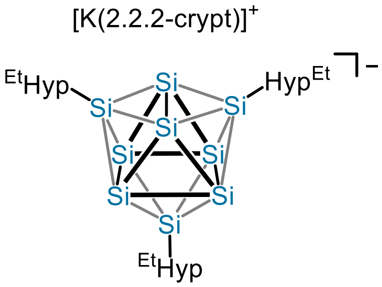

2b

$C_{72}H_{171}KN_2O_6Si_{21}$

1790.03 g/mol

K[K(2.2.2-crypt)]$_2$[Si_9H] (500 mg, 445 µmol, 1.00 eq.) and ^{Et}HypCl (457 mg, 1.38 µmol, 3.10 eq.) were reacted in 10.0 mL THF. After workup, 316 mg (347 µmol, 78%) of [K(2.2.2-crypt)][$^{Et}Hyp_3Si_9$] (**2b**) were obtained as an orange-brown powder. **^{1}H NMR** (500 MHz, thf-d_8, 298 K): δ 0.96 (br q, ^3J(^{1}H, ^{1}H) = 7.8 Hz, 54H, Si(C*H*$_2$CH$_3$)$_3$), 1.06 (br t, ^3J(^{1}H, ^{1}H) = 7.8 Hz, 81H, Si(CH$_2$C*H*$_3$)$_3$), 2.64 (t, 12H, 2.2.2-crypt), 3.63 (t, 12H, 2.2.2-crypt), 3.67 (s, 12H, 2.2.2-crypt); **^{29}Si{^{1}H} IG NMR** (99.4 MHz, thf-d_8, 298 K): δ –352.8 (s, ^1J(^{29}Si, ^{29}Si) = 42.7 Hz, Si_{Prism}), –171.33 (s, ^1J(^{29}Si, ^{29}Si) = 42.7 Hz, Si_{Cap}), –135.16 (s, *Si*($SiEt_3$)$_3$), –1.51 (s, Si(*Si*Et_3)$_3$); **ESI-MS** (negative mode, 3500 V, 300 °C): *m/z* = 1374.45 ([(^{Et}Hyp)$_3Si_9$]$^-$).

[K(2.2.2-crypt)][(^{Me}HypMe$_2$Si)$_3$Si$_9$] (**2c**).

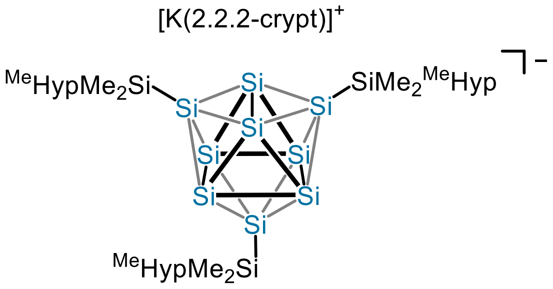

2c

$C_{51}H_{135}KN_2O_6Si_{24}$

1585.78 g/mol

K[K(2.2.2-crypt)]$_2$[Si$_9$H] (500 mg, 445 µmol, 1.00 eq.) and ^{Me}HypMe$_2$SiCl (471 mg, 1.38 mol, 3.10 eq.) were reacted in 10.0 mL THF. After workup, 419 mg (264 µmol, 59%) of [K(2.2.2 crypt)][(^{Me}HypMe$_2$Si)$_3$Si$_9$] (**2c**) were obtained as a brown powder. **^{1}H NMR** (400 MHz, thf-d_8, 298 K): δ 0.28 (s, 81H, ^{Me}Hyp), 0.48 (s, 18H, SiMe$_2$), 2.61 (t, 12H, 2.2.2-crypt), 3.60 (t, 12H, 2.2.2-crypt), 3.64 (s, 12H, 2.2.2-crypt); **^{13}C{^{1}H} NMR** (126 MHz, THF-d_8, 298 K): δ 4.27 (s, ^{Me}Hyp), 9.31 (s, SiMe$_2$), 55.07 (s, 2.2.2-crypt), 67.76 (s, 2.2.2-crypt), 71.62 (s, 2.2.2-crypt); **^{29}Si{^{1}H} IG NMR** (99.4 MHz, THF-d_8, 298 K): δ –347.4 (s, ^1J(^{29}Si, ^{29}Si) = 24.4 Hz, Si$_{Prism}$), –146.30 (s, ^1J(^{29}Si, ^{29}Si) = 24.4 Hz, Si$_{Cap}$), –128.33 (s, *Si*(SiMe$_3$)$_3$), –20.89 (s, SiMe$_2$), –9.94 (s, Si(*Si*Me$_3$)$_3$); **ESI-MS** (negative mode, 3500 V, 300 °C): *m/z* = 1170.19 ([(^{Me}HypMe$_2$Si)$_3$Si$_9$]$^-$).

[K(2.2.2-crypt)][^{t}Bu$_2$FSi)$_3$Si$_9$] (**2d**).

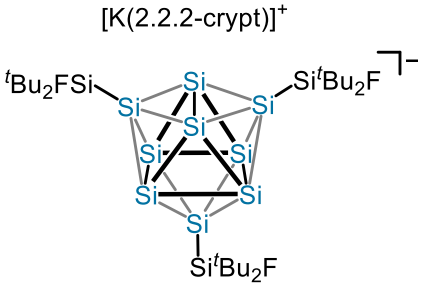

2d

$C_{42}H_{90}F_3KN_2O_6Si_{12}$

1152.29 g/mol

K[K(2.2.2-crypt)]$_2$[Si$_9$H] (500 mg, 445 µmol, 1.00 eq.) and ^{t}Bu$_2$FSiCl (271 mg, 1.38 µmol, 3.10 eq.) were reacted in 10.0 mL THF. After workup, 390 mg (338 µmol, 76%) of [K(2.2.2-crypt)][(^{t}Bu$_2$FSi)$_3$Si$_9$] (**2d**) were obtained as a dark-brown powder. **^{1}H NMR** (500 MHz, thf-d_8, 298 K): δ 1.13 (d, ^3J(^{1}H, ^{19}F) = 1.1 Hz, 18H, ^{t}Bu$_2$FSi), 2.64 (t, 12H, 2.2.2-crypt), 3.63 (t, 12H, 2.2.2-crypt), 3.68 (s, 12H, 2.2.2-crypt); **^{13}C{^{1}H} NMR** (126 MHz, thf-d_8, 298 K): δ 24.21 (d, ^2J(^{19}F, ^{13}C) = 11.0 Hz, C(*C*H$_3$)), 55.02 (s, 2.2.2-crypt), 67.72 (s, 2.2.2-crypt), 71.58 (s, 2.2.2-crypt); **^{19}F{^{1}H} NMR** (470 MHz, thf-d_8, 298 K): δ –171.01 (s, ^{t}Bu$_2$*F*Si); **^{29}Si{^{1}H} IG NMR** (99.4 MHz, thf-d_8, 298 K): δ –357.38 (s, ^1J(^{29}Si, ^{29}Si) = 23.2 Hz, Si$_{Prism}$), –177.67 (d, ^2J(^{19}F, ^{29}Si) = 19.4 Hz, ^1J(^{29}Si, ^{29}Si) = 23.2 Hz, Si$_{Cap}$), 58.97 (d, ^1J(^{19}F, ^{29}Si) = 340.9 Hz, ^{t}Bu$_2$FSi); **ESI-MS** (negative mode, 3500 V, 300 °C): *m/z* = 736.71 ([(^{t}Bu$_2$FSi)Si$_9$]$^-$).

2. Single-crystal XRD data

Supplementary Table 1: Crystallographic data of **1** and **2a**.

Compound	$[K(2.2.2\text{-crypt})]_3[Si_9H]\cdot 8.5\ NH_3$ (**1**)	$[K(2.2.2\text{-crypt})][^{Me}Hyp_3Si_9]\cdot thf$ (**2a**)
Formula	$C_{54}H_{134.5}K_3N_{14.5}O_{18}Si_9$	$C_{49}H_{125}KN_2O_7Si_{21}$
Molecular weight / g·mol^{-1}	1646.37	1483.49
Space group (no.)	$P\bar{1}$ (2)	$P2_1/n$ (14)
a / Å	14.114(1)	15.0913(4)
b / Å	15.1523(9)	24.7859(6)
c / Å	22.903(1)	23.9571(6)
α / deg	80.101(5)	90
β / deg	84.774(5)	90.959(2)
γ / deg	66.454(5)	90
V / Å^3	4421.9(5)	8959.9(4)
Z	2	4
T / K	150(2)	
λ / Å	0.71073	
ρ_{calc} / g·cm^{-3}	1.236	1.100
μ / mm^{-1}	0.340	0.378
Collected reflections	85393	122448
Independent reflections	25771	14496
R_{int} / R_σ	0.023/0.029	0.043 /0.028
Parameters/ restraints	983/100	930/388
R_1 [$I > 2\sigma(I)$ / all data]	0.044/0.067	0.053/0.083
wR_2 [$I > 2\sigma(I)$ / all data]	0.106/0.117	0.119/0.136
Goodness of fit	1.041	1.039
Max. / min. diff. electron density / e·Å^3	–0.58/0.92	–0.47/0.68
CCDC	2338275	2232604

2.1 [K(2.2.2-crypt)]$_3$[Si$_9$H]·8.5NH$_3$ (1) (CCDC 2338275)

Supplementary Table 2: Fractional atomic coordinates, isotropic or equivalent isotropic displacement parameters (Å^2) and side occupancy factors of all cluster positions of **1** (CCDC 2338275).

Atom	*x*	*y*	*z*	U_{iso}*/U_{eq}	Occup. (<1)
**Si1A_1**	0.27954(10)	0.8783(2)	0.74994(4)	0.0276(4)	0.665(8)
**Si1B_1**	0.2673(2)	0.9121(4)	0.75204(9)	0.0310(6)*	0.335(8)
**Si2A_1**	0.3598(6)	0.7550(4)	0.8006(4)	0.0366(11)*	0.205(9)
**Si2B_1**	0.38132(12)	0.74475(8)	0.81666(10)	0.0343(3)	0.795(9)
**Si3A_1**	0.3459(6)	0.6893(10)	0.7385(3)	0.0296(15)*	0.134(10)
**Si3B_1**	0.35218(6)	0.6595(2)	0.73574(3)	0.0295(3)	0.866 (10)
**Si4_1**	0.24048(4)	0.81635(4)	0.67364(2)	0.03405(11)	1
**Si5_1**	0.10147(4)	0.89723(3)	0.74497(2)	0.03137(10)	1
**Si6_1**	0.19832(4)	0.84716(3)	0.84526(2)	0.02931(10)	1
**Si7_1**	0.26279(4)	0.65906(3)	0.83284(2)	0.03298(11)	1
**Si8_1**	0.16589(4)	0.70963(4)	0.73243(2)	0.03067(10)	1
**Si9_1**	0.08325(4)	0.77330(4)	0.82382(3)	0.03695(12)	1

Supplementary Table 3: Atomic displacement parameters (Å^2) of all anisotropically refined cluster positions of **1** (CCDC 2338275).

Atom	U^{11}	U^{22}	U^{33}	U^{12}	U^{13}	U^{23}
**Si1A_1**	0.0296(4)	0.0292(9)	0.0254(4)	−0.0142(5)	0.0008(3)	−0.0015(3)
**Si2B_1**	0.0289(5)	0.0347(4)	0.0361(6)	−0.0068(3)	−0.0102(4)	−0.0062(3)
**Si3B_1**	0.0262(3)	0.0301(7)	0.0275(3)	−0.0069(3)	0.0032(2)	−0.0045(3)
**Si4_1**	0.0428(3)	0.0310(2)	0.0269(2)	−0.0149(2)	0.0038(2)	−0.00155(18)
**Si5_1**	0.0260(2)	0.0275(2)	0.0352(2)	−0.00425(18)	−0.00598(19)	−0.00354(18)
**Si6_1**	0.0397(3)	0.0254(2)	0.0224(2)	−0.01216(19)	0.00297(18)	−0.00562(16)
**Si7_1**	0.0438(3)	0.0230(2)	0.0248(2)	−0.0082(2)	0.00431(19)	0.00058(17)
**Si8_1**	0.0320(3)	0.0329(2)	0.0327(2)	−0.0177(2)	−0.00085(19)	−0.00685(19)
**Si9_1**	0.0333(3)	0.0329(2)	0.0471(3)	−0.0166(2)	0.0157(2)	−0.0118(2)

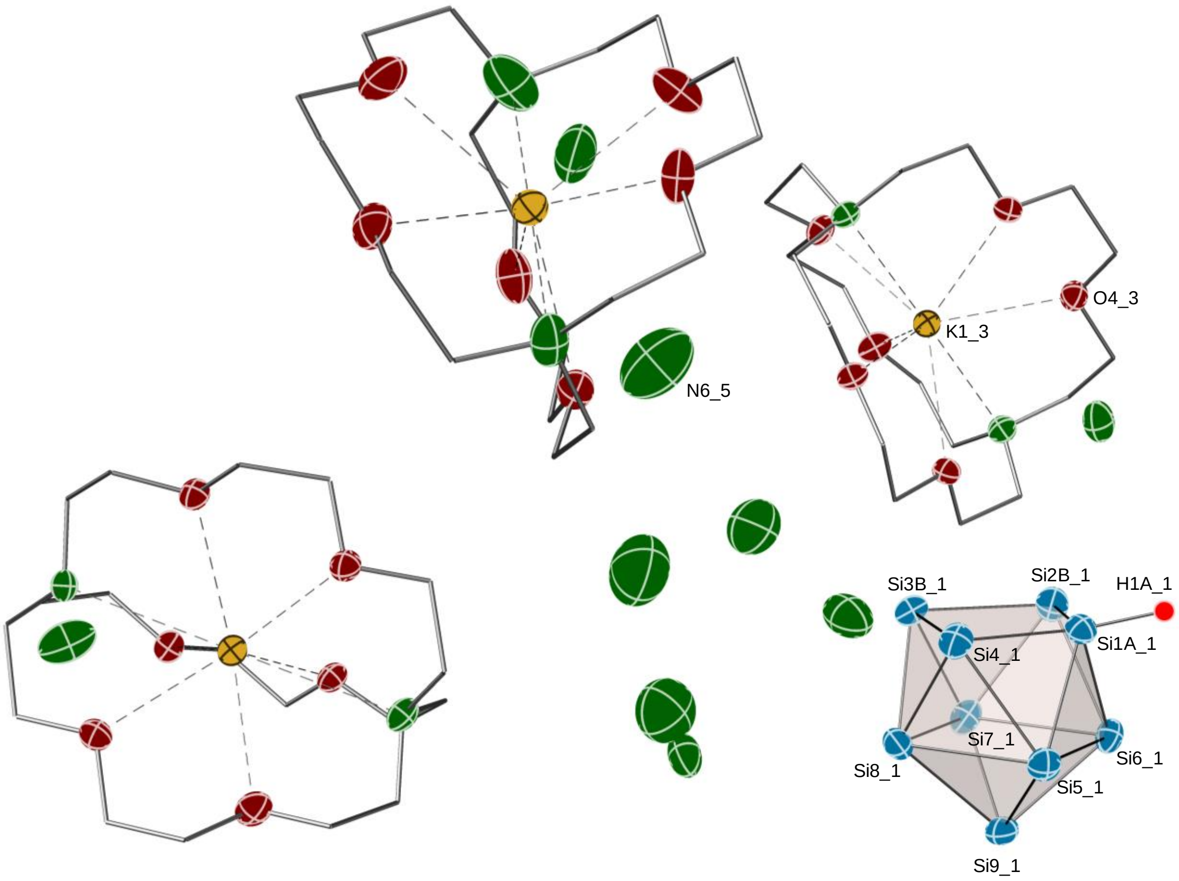

Supplementary Fig. 1: Asymmetric unit of $[K(2.2.2\text{-crypt})]_3[Si_9H]\cdot 8.5NH_3$ (**1**) (CCDC 2338275). Silicon is depicted as blue, oxygen as dark red, nitrogen as green, and potassium as yellow ellipsoids at a 50% probability level. Carbon is depicted as grey wires, H1A_1 as a red sphere of an arbitrary radius, and the remaining hydrogen atoms are omitted for clarity.

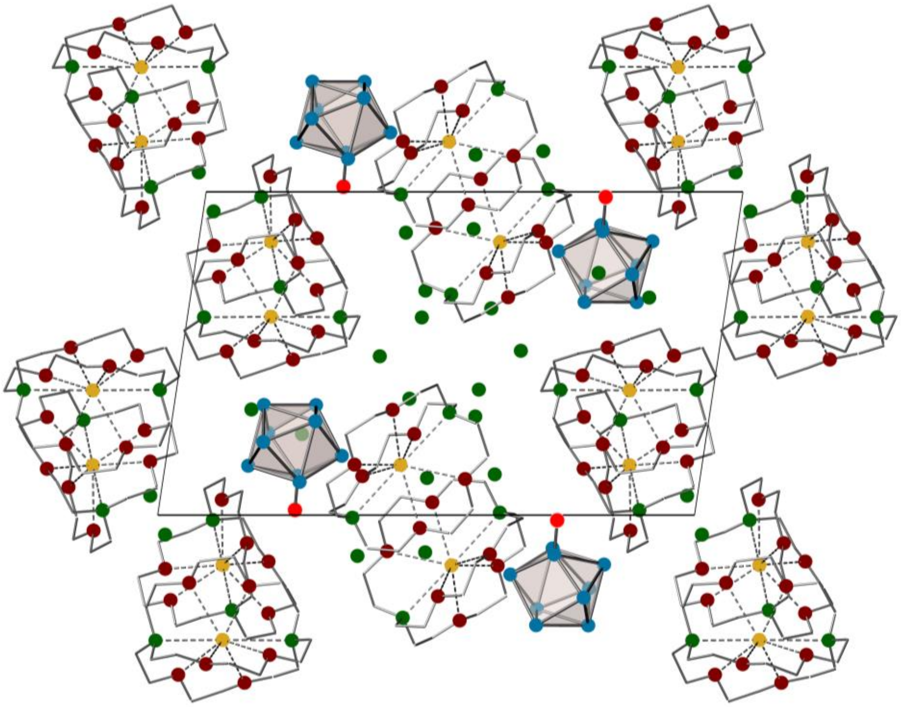

Supplementary Fig. 2: Unit cell of $[K(2.2.2\text{-crypt})]_3[Si_9H]\cdot 8.5NH_3$ (**1**) (CCDC 2338275) in *a* direction. Silicon is depicted as blue, oxygen as dark red, nitrogen as green, potassium as yellow and cluster attached hydrogen atoms as red spheres of an arbitrary radius. Carbon is depicted as grey wires. Hydrogen atoms bound to carbon and nitrogen are omitted for clarity.

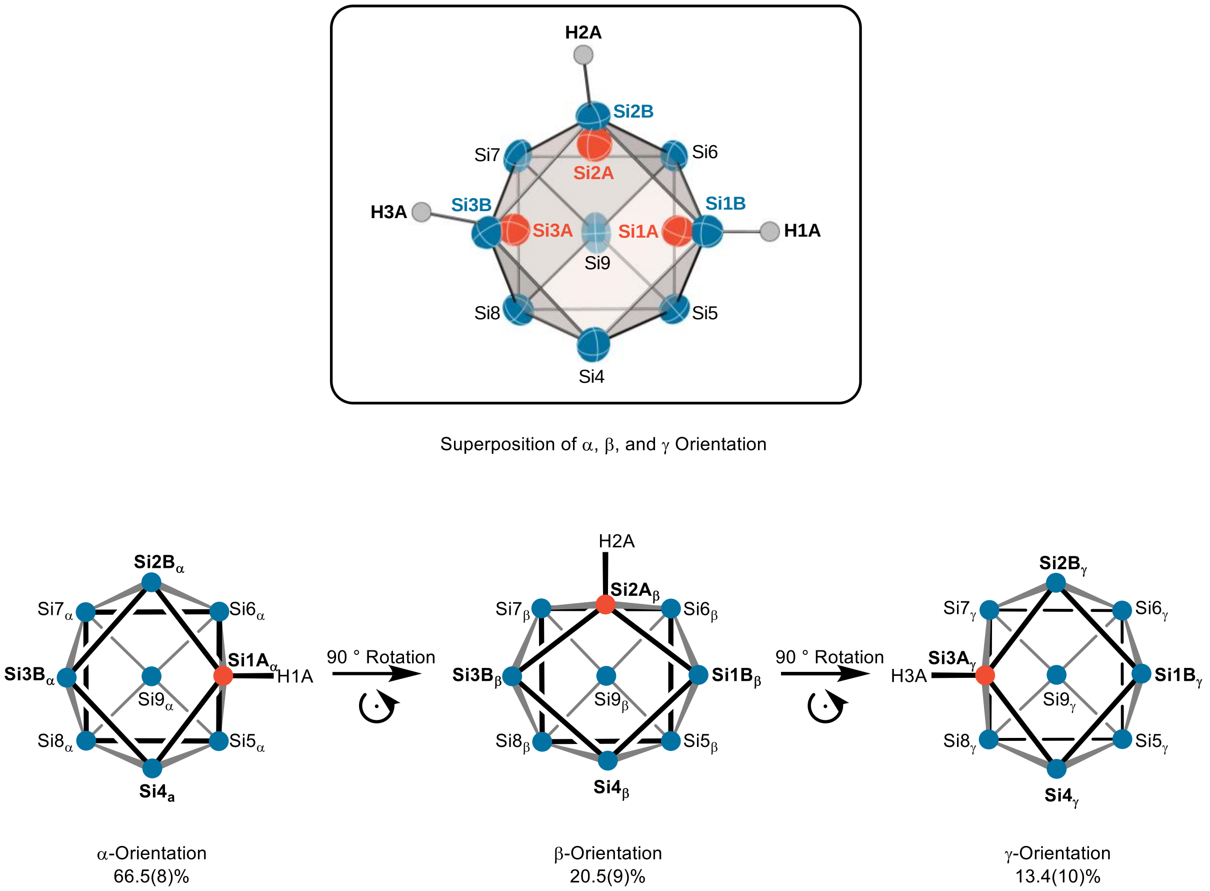

Supplementary Fig. 3: Schematic representation of the orientational disorder of the anionic cluster unit $[Si9H]^{3-}$ in **1** (CCDC 2338275). Protonated positions are depicted in red.

A first refinement resulted in a C_s-symmetric cluster framework distorted on one side along the Si1-Si3 diagonal and exhibiting an elongated Si1 ellipsoid. The one-sided shrinking is a characteristic feature of monosubstituted $[RE_9]^{3-}$ clusters. While other examples of monosubstituted clusters do not exhibit elongated ellipsoids, *Sevov* et al. observed identical elongation of individual ellipsoids of the open square plane in $[Si_9]^{2-}$ and $[Si_9]^{3-}$. However, the obtained charge state of $[Si_9]^{3-}$ was attributed to an oxidation leading to a paramagnetic species.[2] In our case, however, we were able to describe this structural peculiarity as an orientational disorder of different C_s-symmetric orientations (α, β, and γ) in the crystal, resulting from the rotation of the open square plane (Si1-Si2-Si3-Si4) by 90° (Supplementary Fig. 3). Each orientation exhibits a shrunk, protonated SiA position and three naked SiB positions. The occupancies of the SiA positions in the crystal reflect the proportions of individual orientations. Thus, the α-orientation is significantly favoured (66.5(8)%) compared to the others. For this reason, the hydrogen atom H1A at Si1A can be directly refined based on the difference Fourier map. However, this is impossible for the remaining Si2A (β-orientation; 20.5(9)%) and Si3A (γ-orientation; 13.4(10)%) positions. While the geometric influence can be clearly observed based on the one-sided contraction of

each orientation's open square plane, the contributions of β and γ in the structure are too low for a direct refinement of H2A and H3A, respectively. However, H2A and H3A were fixed by SADI instructions based on the Si1A-H1A unit.

The occupancies of all ammonia nitrogen atoms were initially refined freely. The refinement revealed that all nitrogen positions from N1_5 to N8_5 are fully occupied. In contrast, N9_5 shows an occupancy of 50% due to its location near the inversion centre. The elevated anisotropic displacement parameters of some nitrogen atoms are due to a certain degree of disorder of the respective ammonia molecules. Non-coordinated ammonia, similar to water, often exhibits significant disorder. Due to this disorder, we can only refine the hydrogen atoms of ammonia molecules that are coordinated *via* hydrogen bonds and are, therefore, well-localised. For N3_5, we were able to refine a disorder over two positions: N3A_5 (71(3)%) and N3B_5 (29(3)%), respectively.

Check-CIF alerts of **1** (CCDC 2338275):

PLAT910_ALERT_3_B: Missing # of FCF Reflection(s) Below Theta(Min). 14 Note
1 0 0, 0 1 0, 1 1 0, -1 -1 1, 0 -1 1, -1 0 1,
0 0 1, 1 0 1, 0 1 1, 1 1 1, 0 0 2, 1 0 2,
0 1 2, 1 1 2,

Author Response: The low-angle reflections are missing due to the beamstop and large unit cell.

2.2 [K(2.2.2-crypt)][$^{Me}Hyp_3Si_9$]·thf (2a) (CCDC 2232604)

Supplementary Table 4: Fractional atomic coordinates of all cluster positions of **2a** (CCDC 2232604).

Atom	*x*	*y*	*z*
**Si1_1**	0.34666(5)	0.75084(3)	0.52577(3)
**Si2_1**	0.41864(5)	0.83264(3)	0.48545(3)
**Si3_1**	0.26442(5)	0.80707(3)	0.45502(3)
**Si4_1**	0.27813(5)	0.71119(3)	0.44427(3)
**Si5_1**	0.49040(5)	0.74618(3)	0.48407(3)
**Si6_1**	0.37843(5)	0.82467(3)	0.38852(3)
**Si7_1**	0.34097(5)	0.73537(3)	0.35564(3)
**Si8_1**	0.42476(5)	0.67833(3)	0.42447(3)
**Si9_1**	0.49618(5)	0.75920(3)	0.38427(3)

Supplementary Table 5: Atomic displacement parameters (Å^2) of all cluster positions of **2a** (CCDC 2232604).

Atom	U^{11}	U^{22}	U^{33}	U^{12}	U^{13}	U^{23}
**Si1_1**	0.0428(4)	0.0494(4)	0.0428(4)	−0.0053(3)	0.0007(3)	0.0052(3)
**Si2_1**	0.0468(4)	0.0402(4)	0.0473(4)	−0.0048(3)	−0.0025(3)	0.0001(3)
**Si3_1**	0.0404(4)	0.0424(4)	0.0472(4)	0.0013(3)	0.0015(3)	0.0010(3)
**Si4_1**	0.0427(4)	0.0411(4)	0.0489(4)	−0.0065(3)	0.0067(3)	−0.0048(3)
**Si5_1**	0.0406(4)	0.0511(4)	0.0476(4)	0.0042(3)	−0.0014(3)	−0.0030(3)
**Si6_1**	0.0541(4)	0.0405(4)	0.0410(4)	−0.0017(3)	0.0054(3)	0.0032(3)
**Si7_1**	0.0506(4)	0.0466(4)	0.0442(4)	−0.0052(3)	0.0007(3)	−0.0016(3)
**Si8_1**	0.0475(4)	0.0396(4)	0.0596(5)	0.0022(3)	0.0021(3)	−0.0019(3)
**Si9_1**	0.0423(4)	0.0576(5)	0.0506(4)	−0.0027(3)	0.0075(3)	0.0035(4)

Supplementary Table 6: Selected bond length (Å) in **2a** (CCDC 2232604) and related cluster species.

Bond	[$^{Me}Hyp_2Si_9]^{2-}$ [1]	(**2a**)	[$^{Me}Hyp_2Ge_9]^{2-}$ [3]	[$^{Me}Hyp_3Ge_9]^{-}$ [4] [a]
exo ^{Me}Hyp-E4	2.357(5)	2.3315(10)	2.4071(9)	2.3646(14)
exo ^{Me}Hyp- E5	2.339(5)	2.3254(10)	2.4098(9)	2.3741(15)
exo ^{Me}Hyp- E6	-	2.3259(10)	-	2.3688(14)
E1-E4	2.427(5)	2.4041(11)	2.5528(5)	2.5321(8)
E1-E5	2.398(5)	2.4061(11)	2.5469(5)	2.5213(8)
E4-E8	2.396(5)	2.4125(11)	2.5415(5)	2.5366(8)
E5-E8	2.395(4)	2.4086(11)	2.5486(5)	2.5314(8)
E1-E2	2.488(5)	2.5017(10)	2.6439(5)	2.7081(7)
E1-E3	2.485(6)	2.5071(11)	2.6399(5)	2.6526(8)
E2-E3	2.585(6)	2.5086(11)	2.7046(5)	2.6921(8)
E1-E8	3.770(7)	3.2565(10)	3.9867(9)	3.4966(9)
E2-E9	2.738(5)	3.2639(10)	2.8837(5)	3.2196(9)
E3-E7	2.772(6)	3.2021(10)	2.6005(5)	3.4904(9)
E7-E9	2.569(6)	2.5003(11)	2.7076(5)	2.6640(8)
E2-E6	2.475(7)	2.3986(10)	2.5824(5)	2.5234(8)
E3-E6	2.468(6)	2.4043(10)	2.5872(5)	2.5222(7)
E6-E7	2.470(6)	2.4135(11)	2.5751(8)	2.5238(8)
E6-E9	2.427(6)	2.4100(11)	2.5819(5)	2.5343(8)

[a] Two independent clusters in the unit cell.

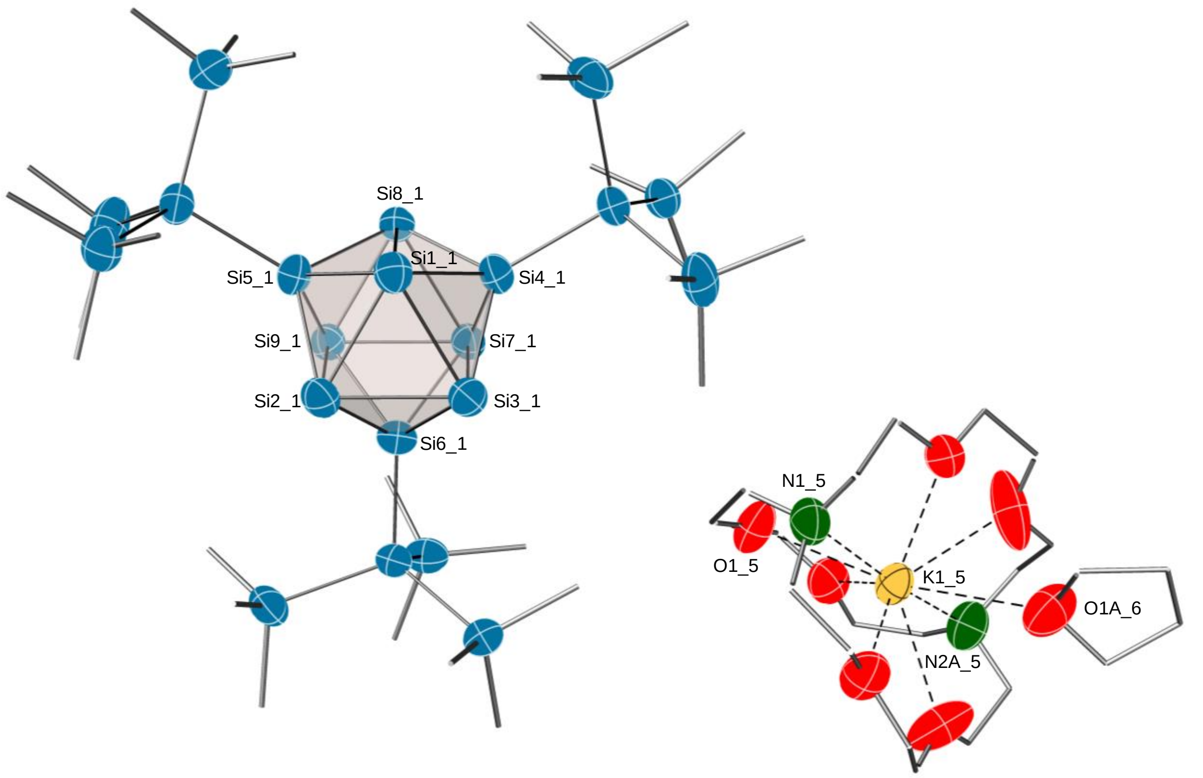

Supplementary Fig. 4: Asymmetric unit of [K(2.2.2-crypt)][$^{Me}Hyp_3Si_9$]·thf (**2a**) (CCDC 2232604). Silicon is depicted as blue, oxygen as red, nitrogen as green, and potassium as yellow ellipsoids at a 50% probability level. Carbon is depicted as grey wires. Hydrogen atoms are omitted for clarity.

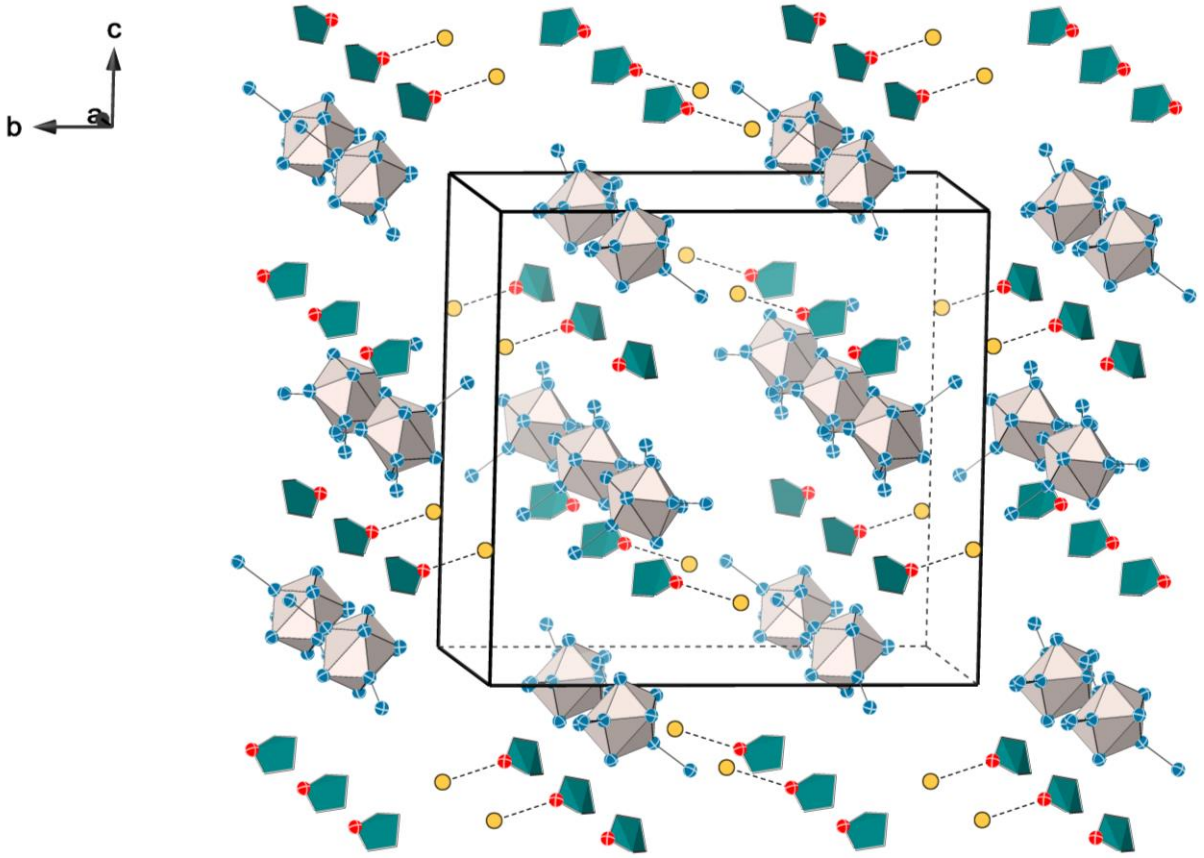

Supplementary Fig. 5: Unit cell of [K(2.2.2-crypt)][^{Me}Hyp$_3$Si$_9$]·thf (**2a**) (CCDC 2232604). Silicon is depicted as blue, oxygen as red, and potassium as yellow spheres of an arbitrary radius. Carbon atoms of thf (visualised as tile rings) are depicted as grey wires. Hydrogen atoms, TMS groups, and 2.2.2-cryptand are omitted for clarity.

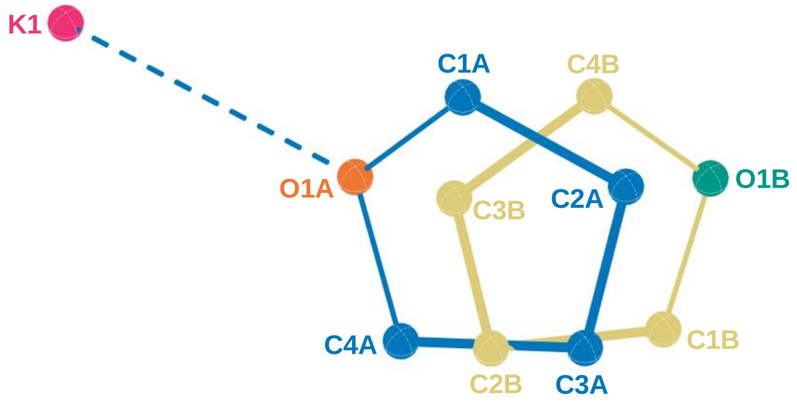

Supplementary Fig. 6: Disorder model of thf in **2a** (CCDC 2232604). Component A (51.1(8)%) is indicated by blue bonds and component B (48.9(8)%) by yellow bonds. All atoms are shown as spheres of an arbitrary radius and hydrogen atoms are omitted for clarity.

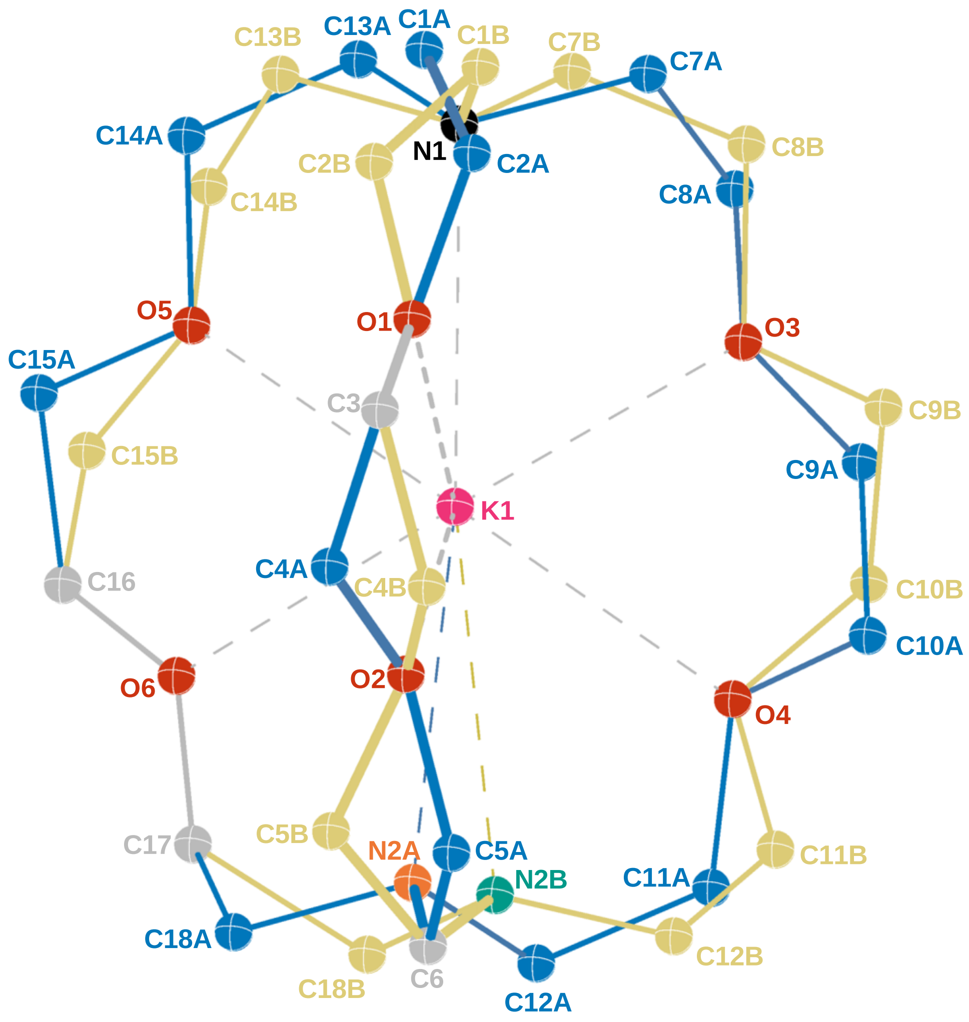

Supplementary Fig. 7: Disorder model of $[K(2.2.2\text{-crypt})]^+$ unit in **2a** (CCDC 2232604). Component A (57.9(6)%) is indicated by blue bonds and component B (42.1(6)%) by yellow bonds. Non-disordered bonds are depicted in grey. All atoms are shown as spheres of an arbitrary radius and hydrogen atoms are omitted for clarity.

The asymmetric unit of **2a** (Supplementary Fig. 4) contains one monoanionic, trisilylated $[^{Me}Hyp_3Si_9]^-$ cluster anion, one $[K(2.2.2\text{-crypt})]^+$ counter cation, and one thf molecule. While the cluster unit is well-ordered, an initial refinement of thf and $[K(2.2.2\text{-crypt})]^+$ revealed large anisotropic displacement parameters (ADPs). However, a split-layer refinement using rigid bond restraints (RIGU) and fixed relative 1,2- and 1,3-distances (SADI) allowed for a satisfactory refinement of both entities.

The disorder model for thf (Supplementary Fig. 6) indicates two different orientations, A (51.1(8)%) and B (48.9(8)%), in the unit cell. In orientation A, O1A_6 coordinates to K1_5 of the neighbouring $[K(2.2.2\text{-crypt})]^+$ ion (O1A_6 - K1_5 = 3.411(6) Å). In orientation B, however, O1B_6 is oriented in the opposite direction, causing a greater distance to $[K(2.2.2\text{-crypt})]^+$ due to the lack of coordination. It is

important to note that orientations A and B are not related by mirror symmetry but represent distinct conformers.

Similar to thf, a split-layer refinement of [K(2.2.2-crypt)]$^+$ reveals an orientational disorder over two conformers, A (57.9(6)%) and B (42.1(6)%), in the unit cell (Supplementary Fig. 7). Starting from the mutual tertiary amino nitrogen atom N1_5, conformer B arises from the rotation of the first methylene groups (C1A_5, C7A_5, and C13A_5) by an average of 27.6° around the N1_5-K1_5 axis (Supplementary Fig. 8a). While most of the remaining methylene groups show splitting (except C3_5, C6_5, C16_5, and C17_5) both conformers share mutual potassium (K1_5) and oxygen positions (O1_5 to O6_5). The splitting of the terminating ternary amino position causes N2A_5 and N2B_5 to tilt relative to the N1_5-K1_5 axis by tilt angles of 6.9(2)° and 7.2(3)°, respectively.

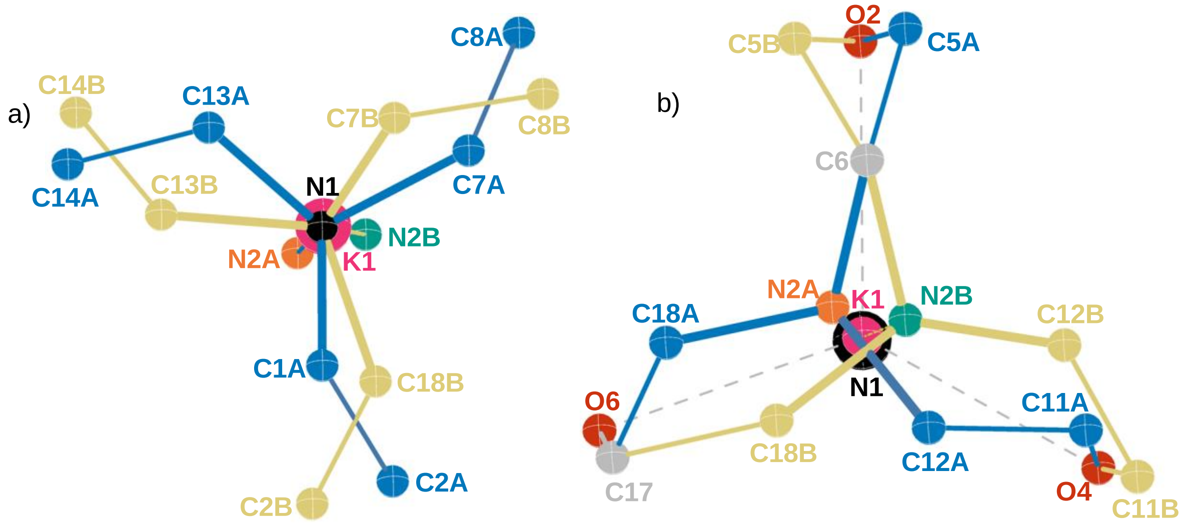

Supplementary Fig. 8: a) Top and b) bottom view along N1-K1 axis of disordered [K(2.2.2-crypt)]$^+$ unit in **2a** (CCDC 2232604). Component A (57.9(6)%) is indicated by blue bonds and component B (42.1(6)%) by yellow bonds. Non-disordered bonds are depicted in grey. All atoms are shown as spheres of an arbitrary radius. Higher coordination spheres and hydrogen atoms are omitted for clarity.

Check-CIF alerts of **2a** (CCDC 2232604):

**PLAT910_ALERT_3_B**:	Missing # of FCF Reflection(s) Below Theta(Min). 16 Note<br>1 1 0, 0 2 0, 1 2 0, -1 0 1, 1 0 1, -1 1 1,<br>0 1 1, 1 1 1, -1 2 1, 0 2 1, 1 2 1, 0 0 2,<br>-1 1 2, 0 1 2, 1 1 2, 0 2 2,
**Author Response:**	The low-angle reflections are missing due to the beamstop and large unit cell.

3. Powder X-ray data

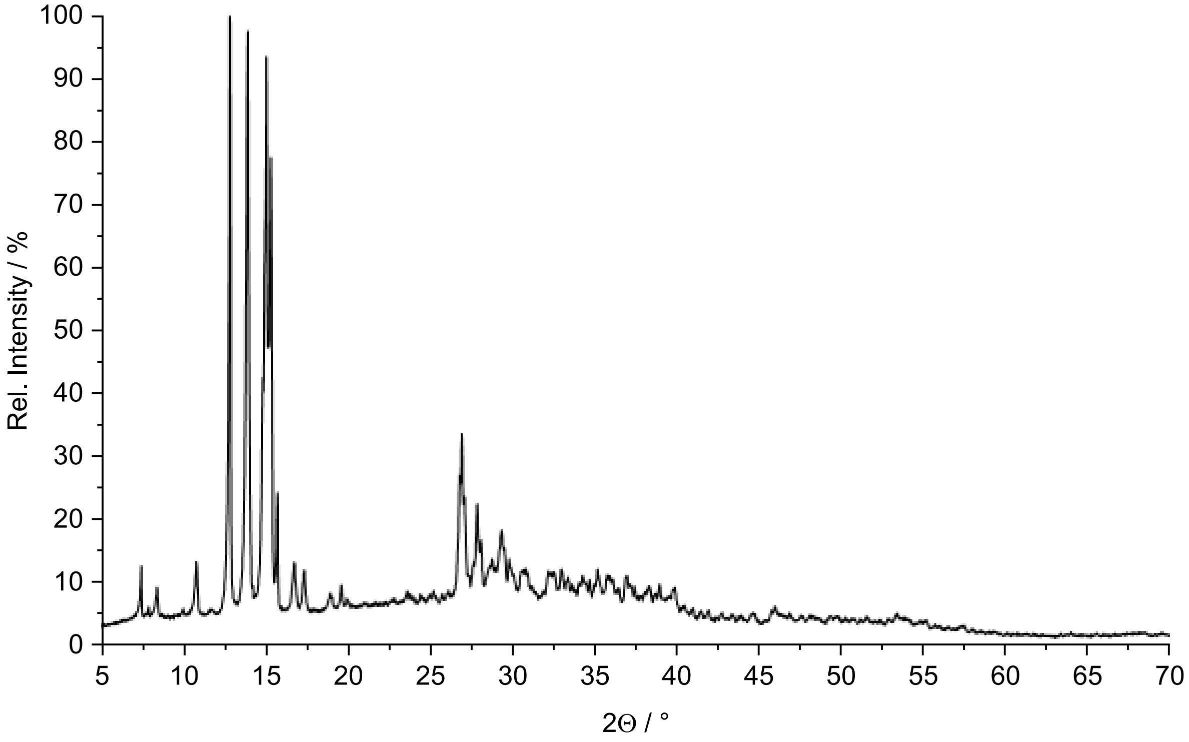

Supplementary Fig. 9: Powder X-ray diffraction pattern (PXRD) of $K_{12}Si_{17}$.

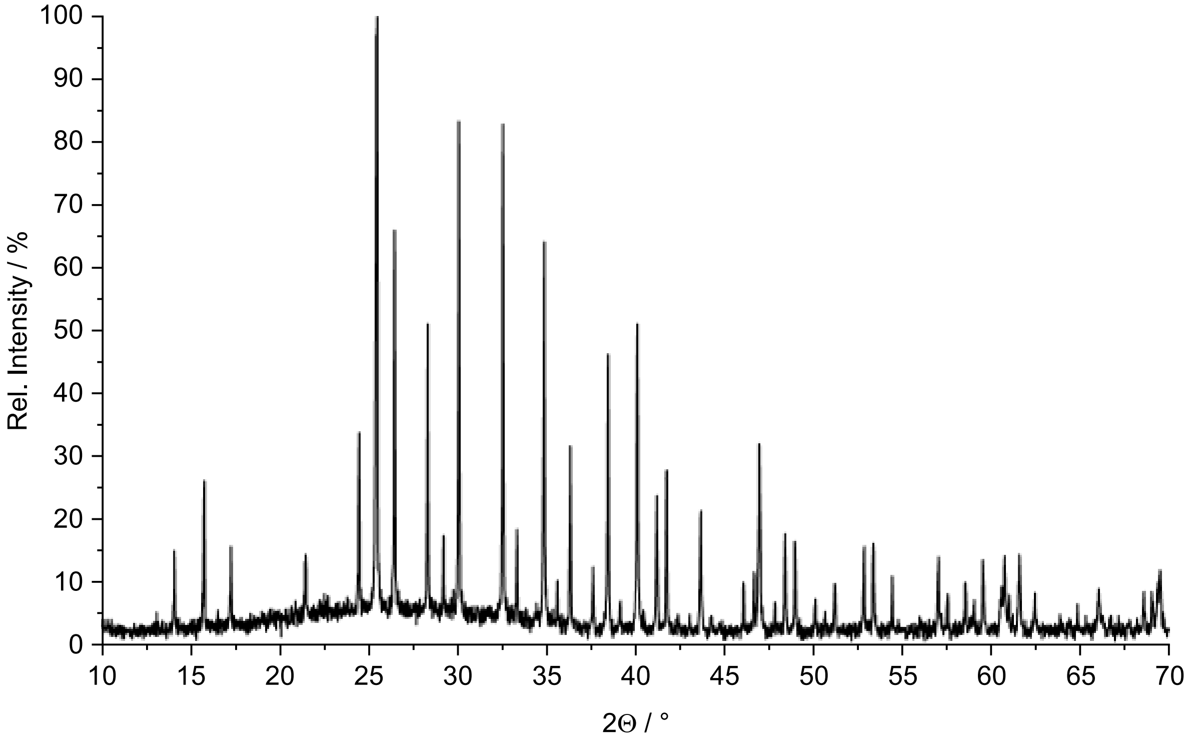

Supplementary Fig. 10: Powder X-ray diffraction pattern (PXRD) of K_4Si_4.

4. NMR data

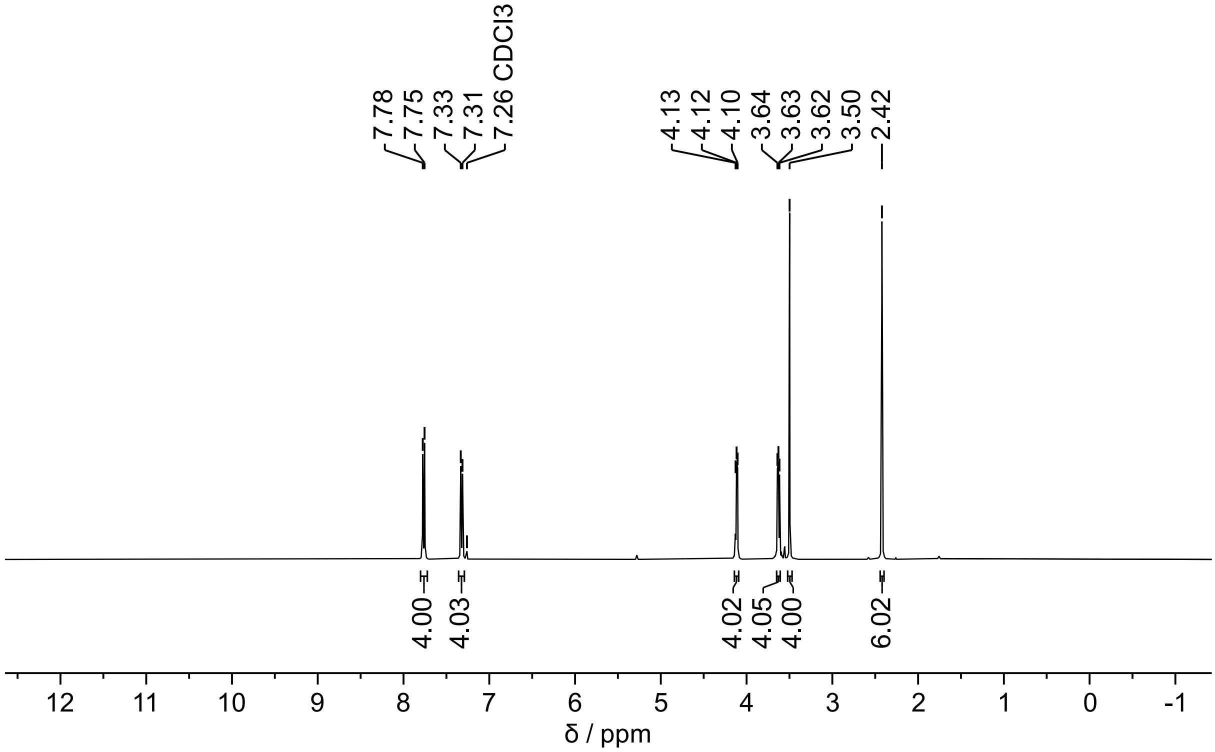

Supplementary Fig. 11: ^{1}H NMR of triethylene glycol bis(*p*-toluenesulfonate) (400 MHz, $CDCl_3$, 298 K).

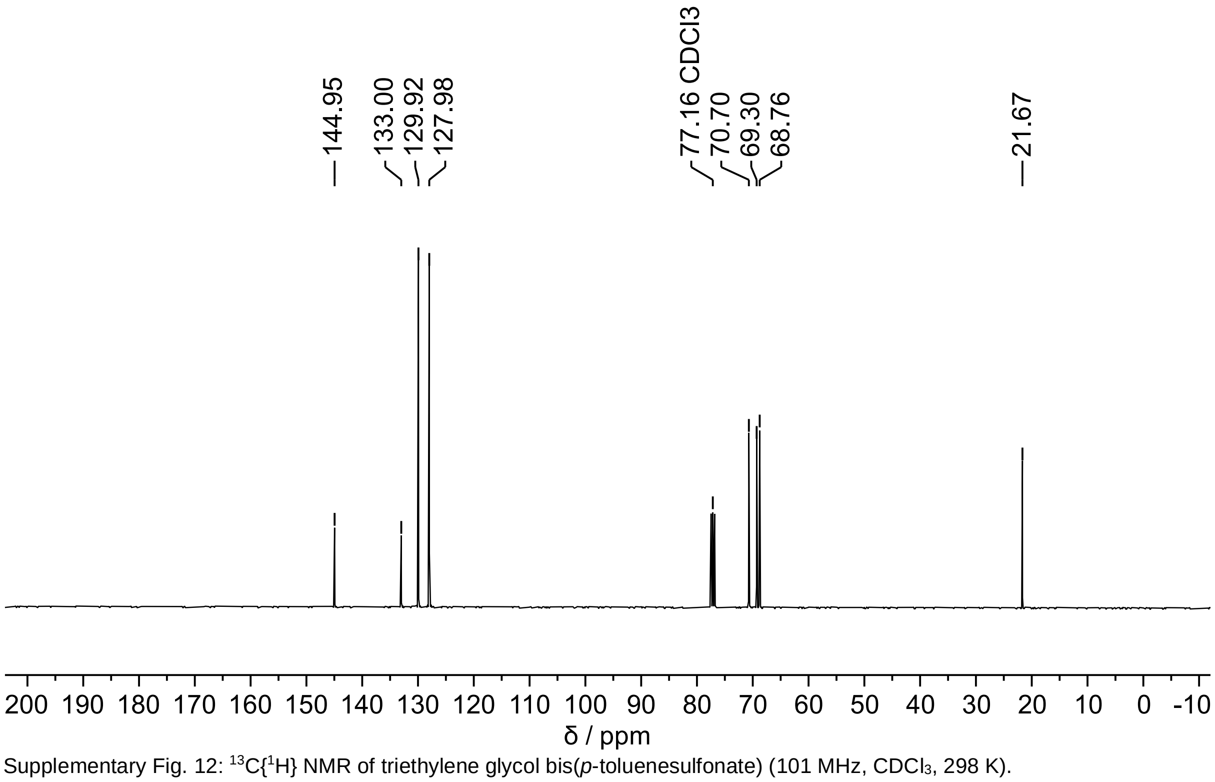

Supplementary Fig. 12: ^{13}C{^{1}H} NMR of triethylene glycol bis(*p*-toluenesulfonate) (101 MHz, $CDCl_3$, 298 K).

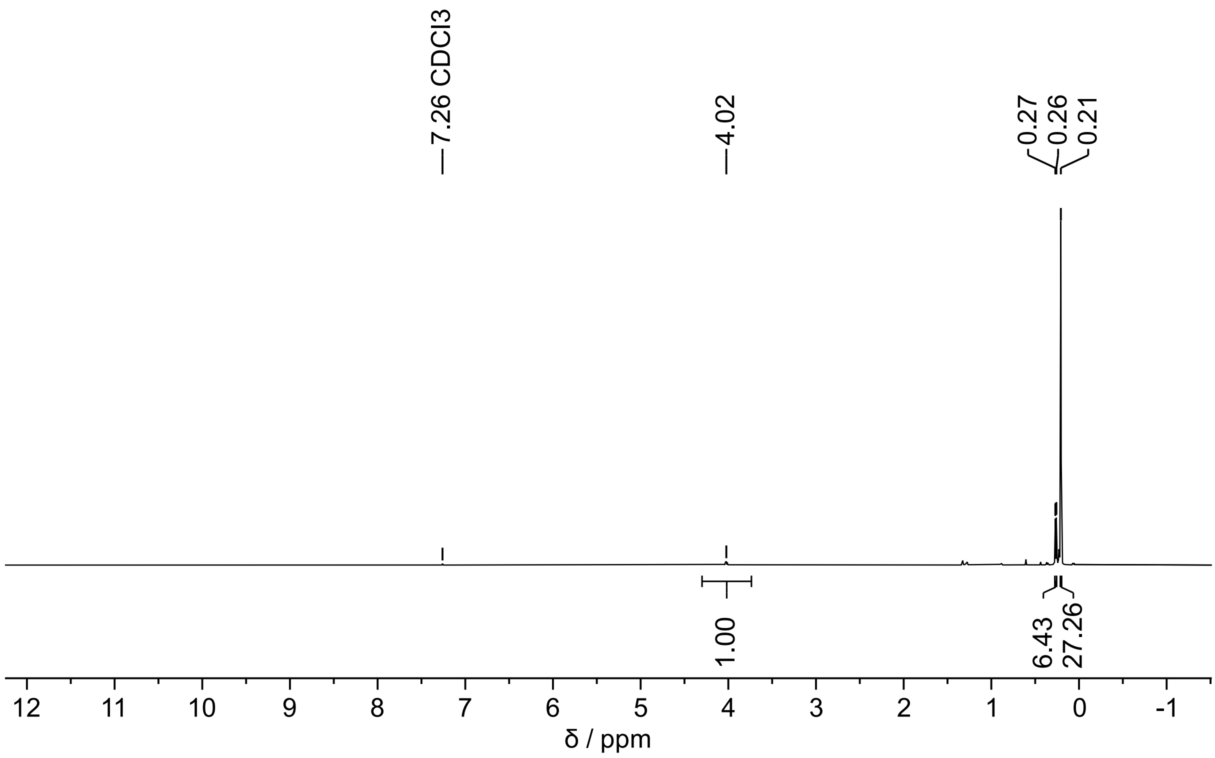

Supplementary Fig. 13: ^{1}H NMR of $^{Me}HypMe_2SiH$ (400 MHz, $CDCl_3$, 298 K).

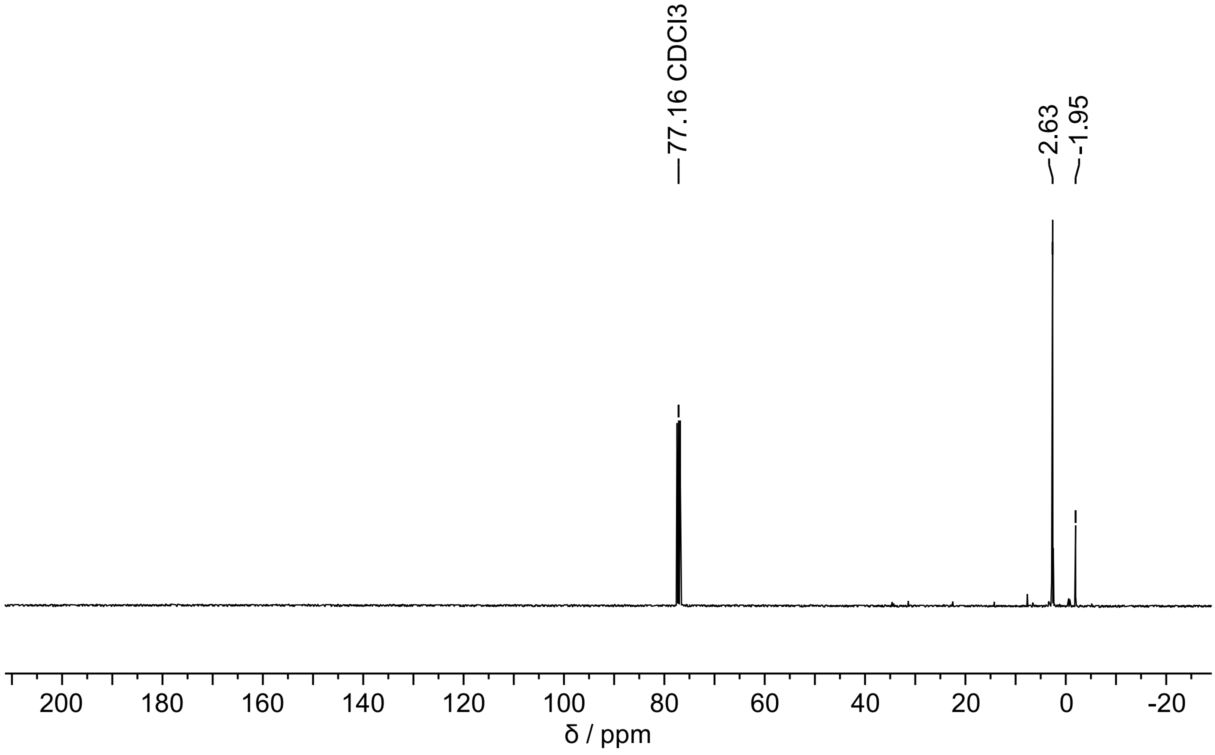

Supplementary Fig. 14: $^{13}C\{^{1}H\}$ NMR of $^{Me}HypMe_2SiH$ (101 MHz, $CDCl_3$, 298 K).

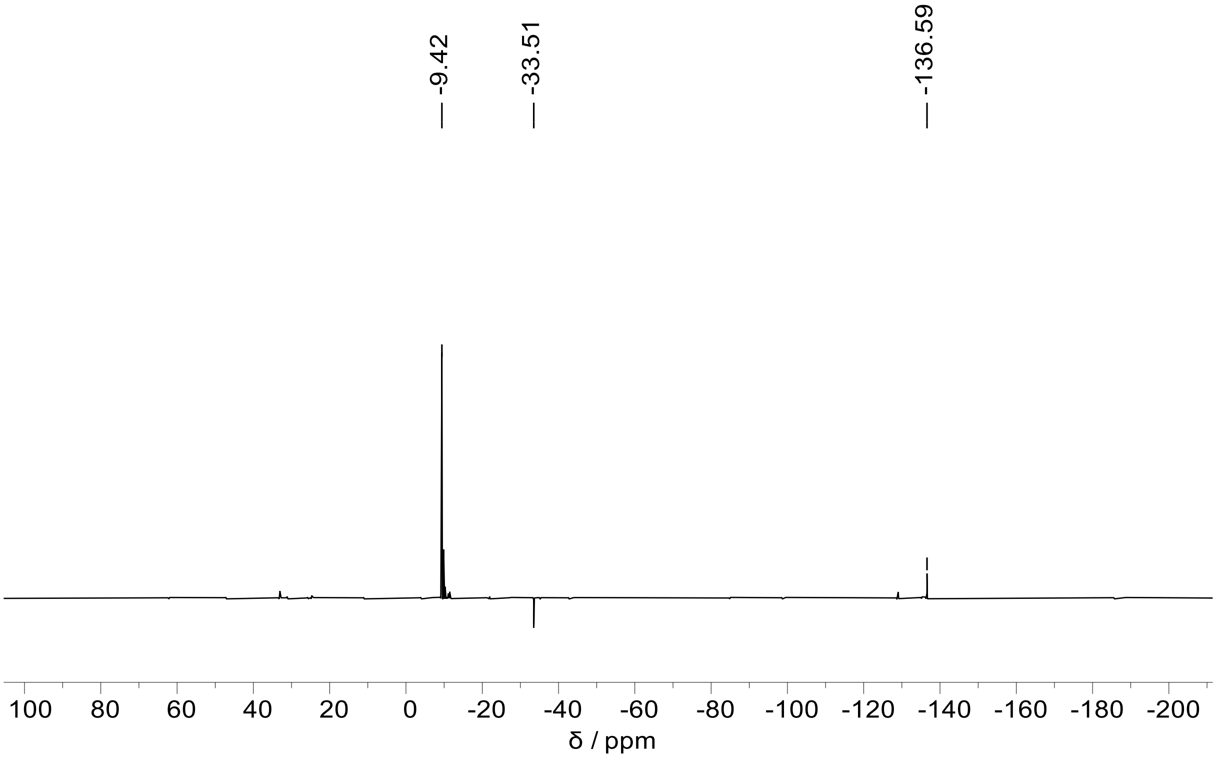

Supplementary Fig. 15: $^{29}Si\{^{1}H\}$ INEPT NMR of $^{Me}HypMe_2SiH$ (79.5 MHz, $CDCl_3$, 298 K).

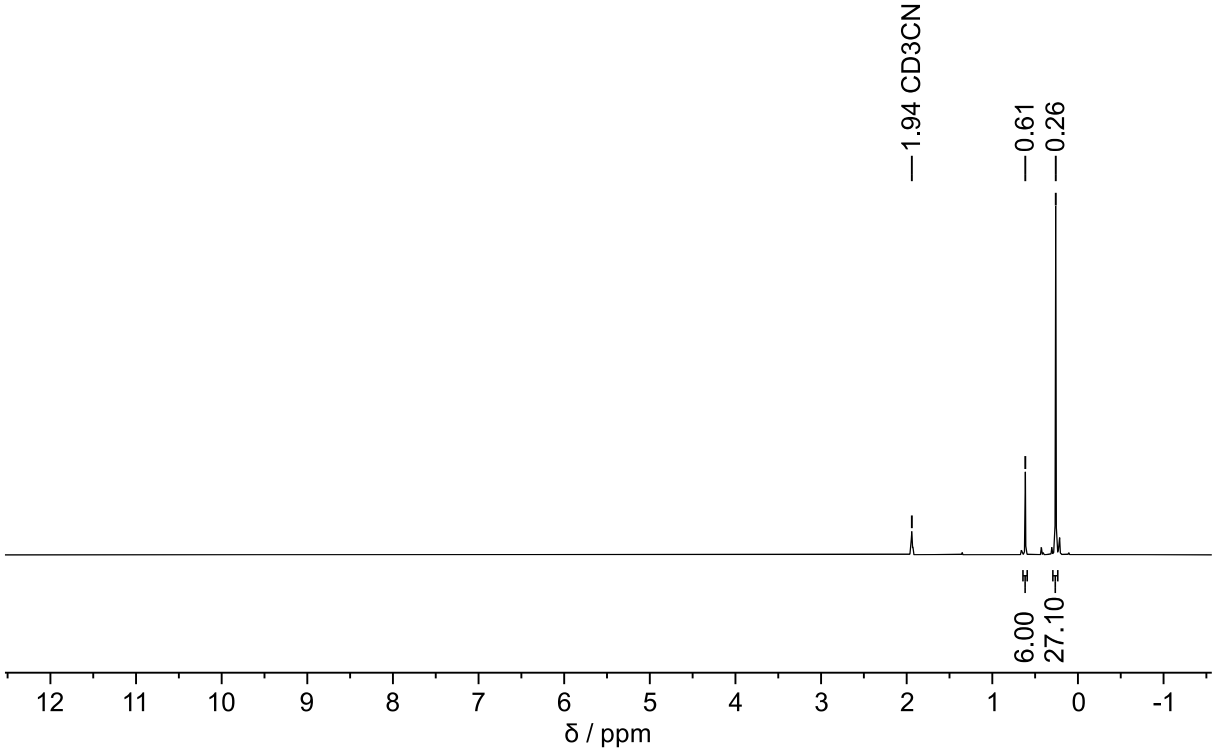

Supplementary Fig. 16: ^{1}H NMR of $^{Me}HypMe_2SiCl$ (400 MHz, $CDCl_3$, 298 K).

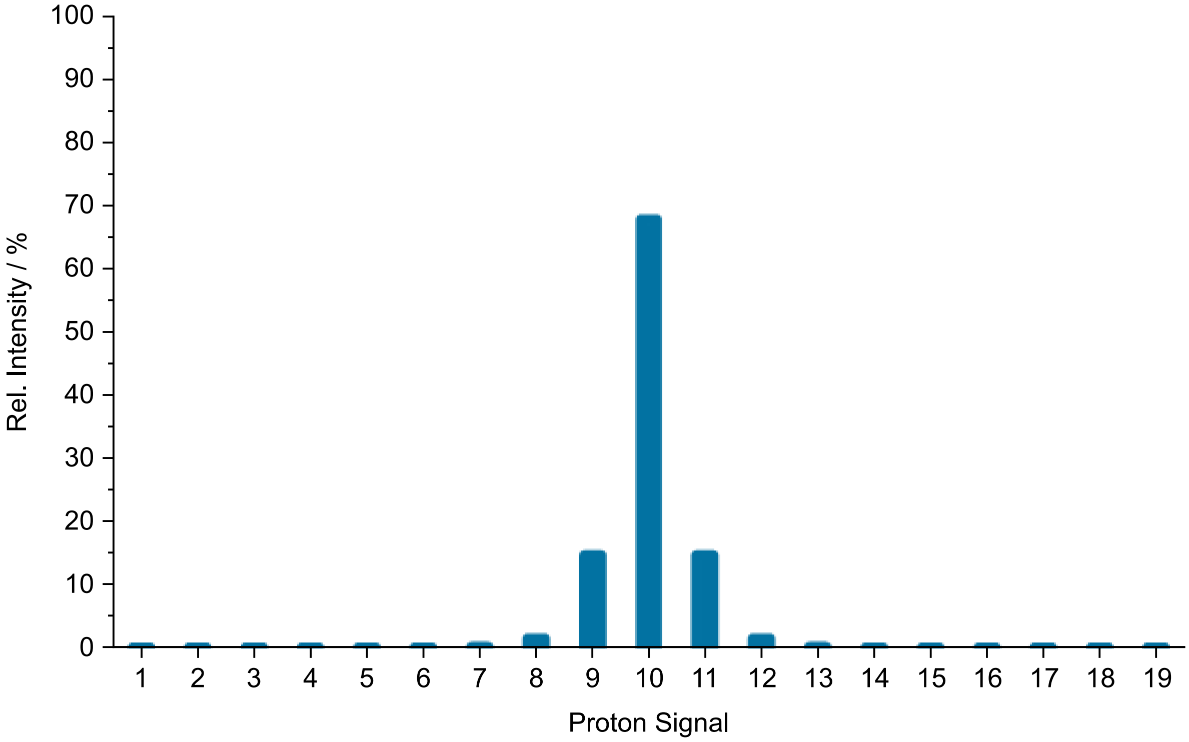

Supplementary Fig. 17: Theoretical ^{1}H NMR satellite pattern for $[Si_9H]^{3-}$ at the natural abundance level of ^{29}Si (NA = 4.7%). Signal 10 reflects the main signal caused by the non-coupling isotopologues $[^{28/30}Si_9H]^{3-}$. Intensities are given in Supplementary Table 7.

Supplementary Table 7: Relative intensities of a theoretical ^{1}H NMR spectrum (Supplementary Fig. 17) of $[Si_9H]^{3-}$ at the natural abundance level of ^{29}Si (NA = 4.7%). Signal 10 reflects the main signal caused by the non-coupling isotopologues $[^{28/30}Si_9H]^{3-}$.

**Proton Signal**	**Rel. Intensity / %**
1	$2.06339 \cdot 10^{-13}$
2	$7.58171 \cdot 10^{-11}$
3	$1.23833 \cdot 10^{-8}$
4	$1.18008 \cdot 10^{-6}$
5	$7.23176 \cdot 10^{-5}$
6	0.00296
7	0.08062
8	1.41619
9	14.57547
10	67.84937
11	14.57547
12	1.41619
13	0.08062
14	0.00296
15	$7.23176 \cdot 10^{-5}$
16	$1.18008 \cdot 10^{-6}$
17	$1.23833 \cdot 10^{-8}$
18	$7.58171 \cdot 10^{-11}$
19	$2.06339 \cdot 10^{-13}$

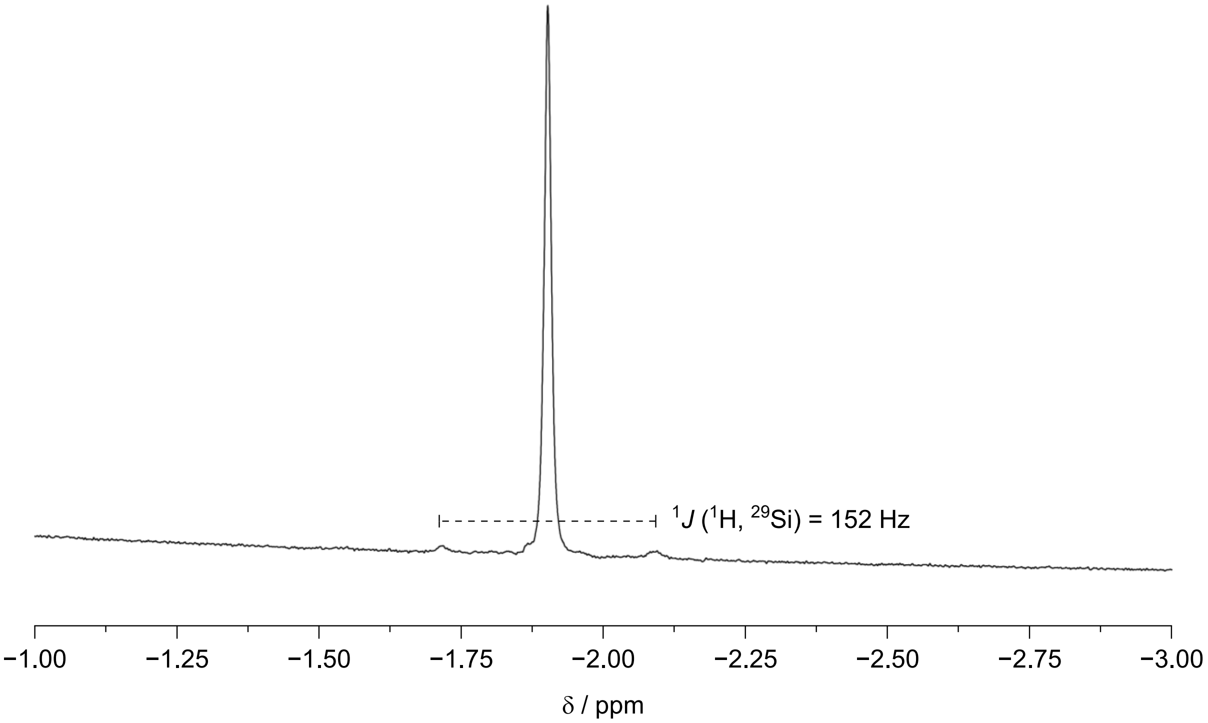

Supplementary Fig. 18: ^{1}H NMR of the dried filtrate (400 MHz, DMF-d_7, 223 K).

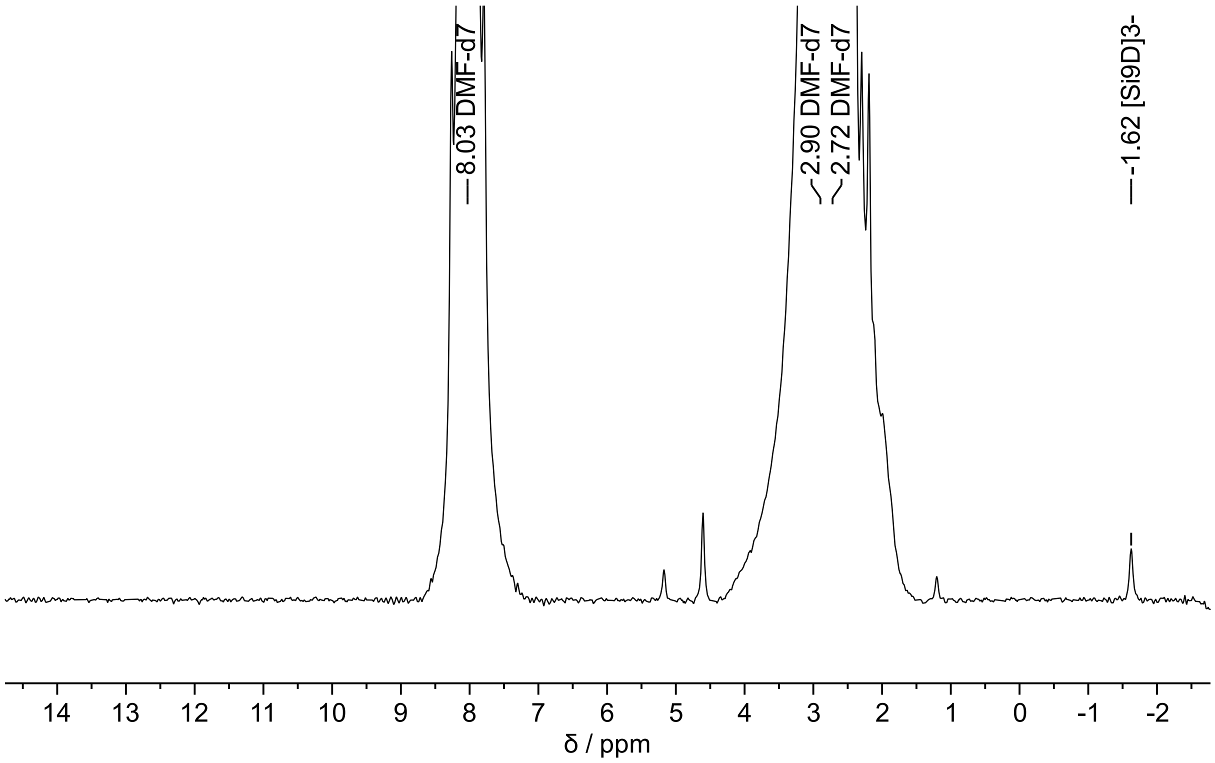

Supplementary Fig. 19: ^{2}H NMR of K[K(2.2.2-crypt)$_2$[Si$_9$D] (61.4 MHz, DMF-d_7, 300 K).

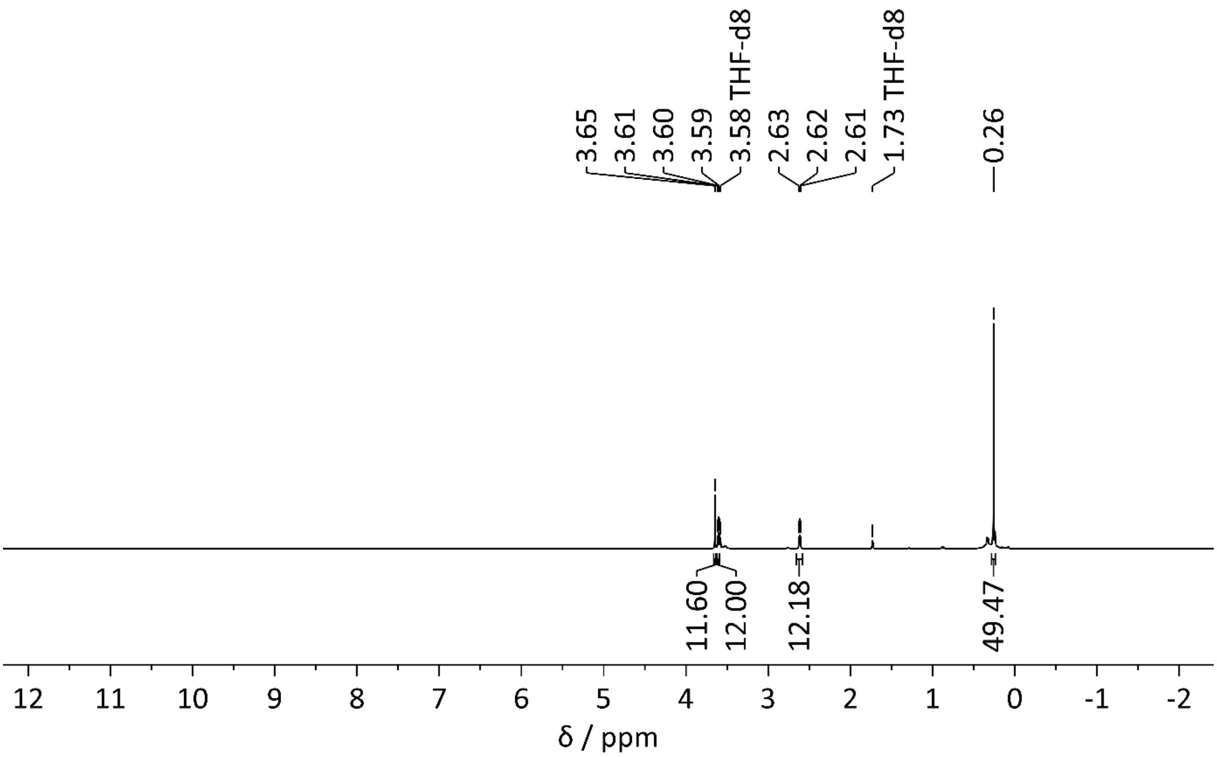

Supplementary Fig. 20: ^{1}H NMR of [K(2.2.2-crypt)][$^{Me}Hyp_3Si_9$] (**2a**) (500 MHz, thf-d_8, 300 K).

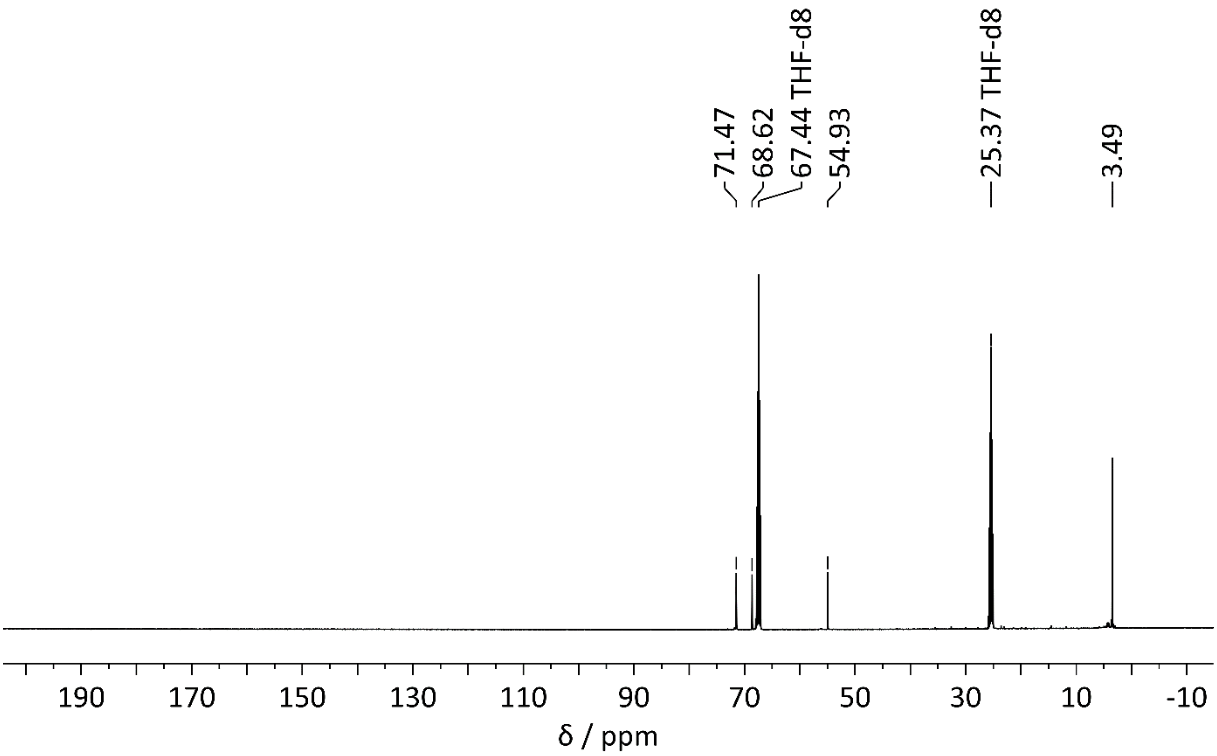

Supplementary Fig. 21: $^{13}C\{^{1}H\}$ NMR of [K(2.2.2-crypt)][$^{Me}Hyp_3Si_9$] (**2a**) (126 MHz, thf-d_8, 300 K).

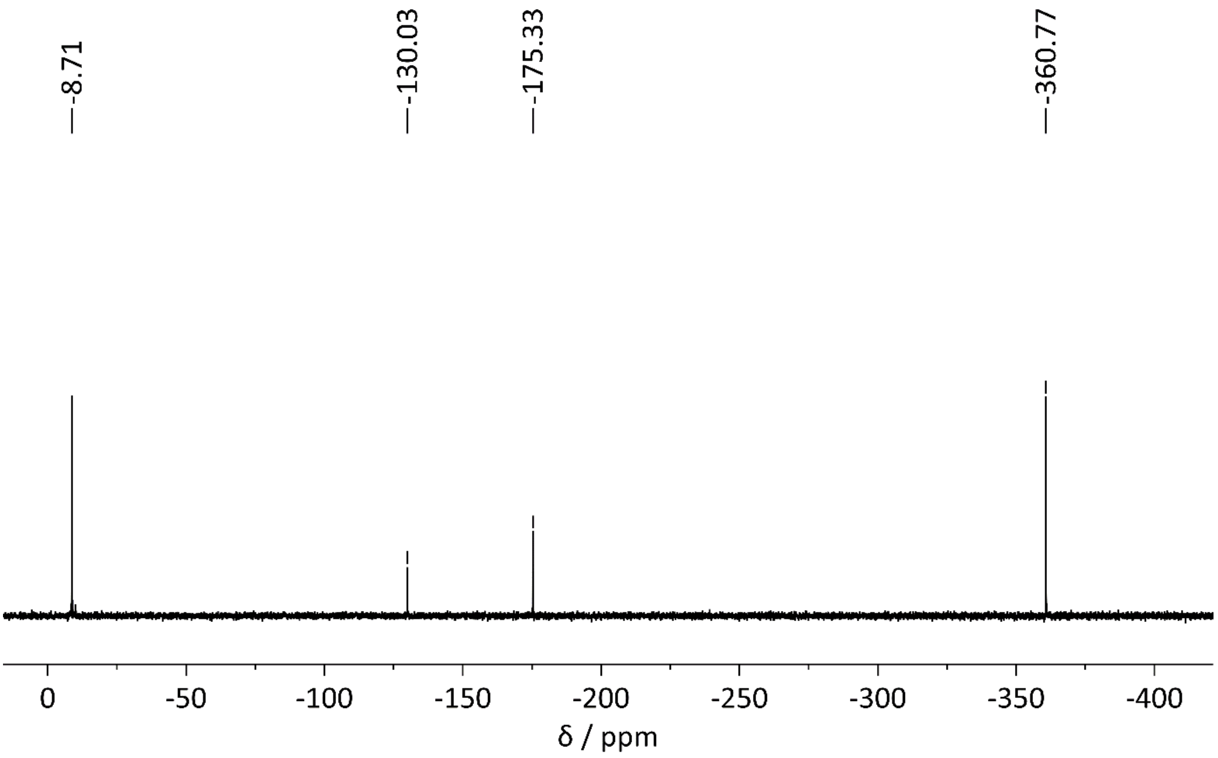

Supplementary Fig. 22: $^{29}Si\{^1H\}$ IG NMR of [K(2.2.2-crypt)][^{Me}Hyp$_3$Si$_9$] (**2a**) (99.4 MHz, thf-d_8, 300 K).

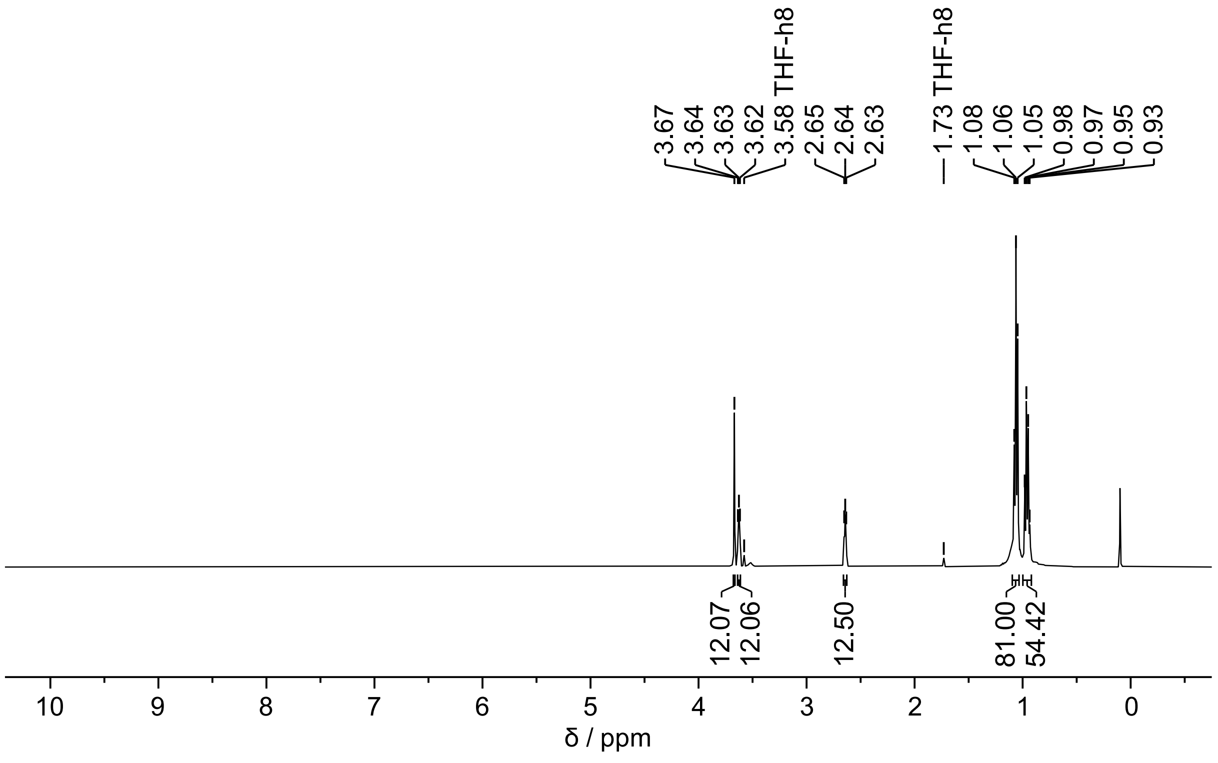

Supplementary Fig. 23: ^1H NMR of [K(2.2.2-crypt)][^{Et}Hyp$_3$Si$_9$] (**2b**) (500 MHz, thf-d_8, 300 K).

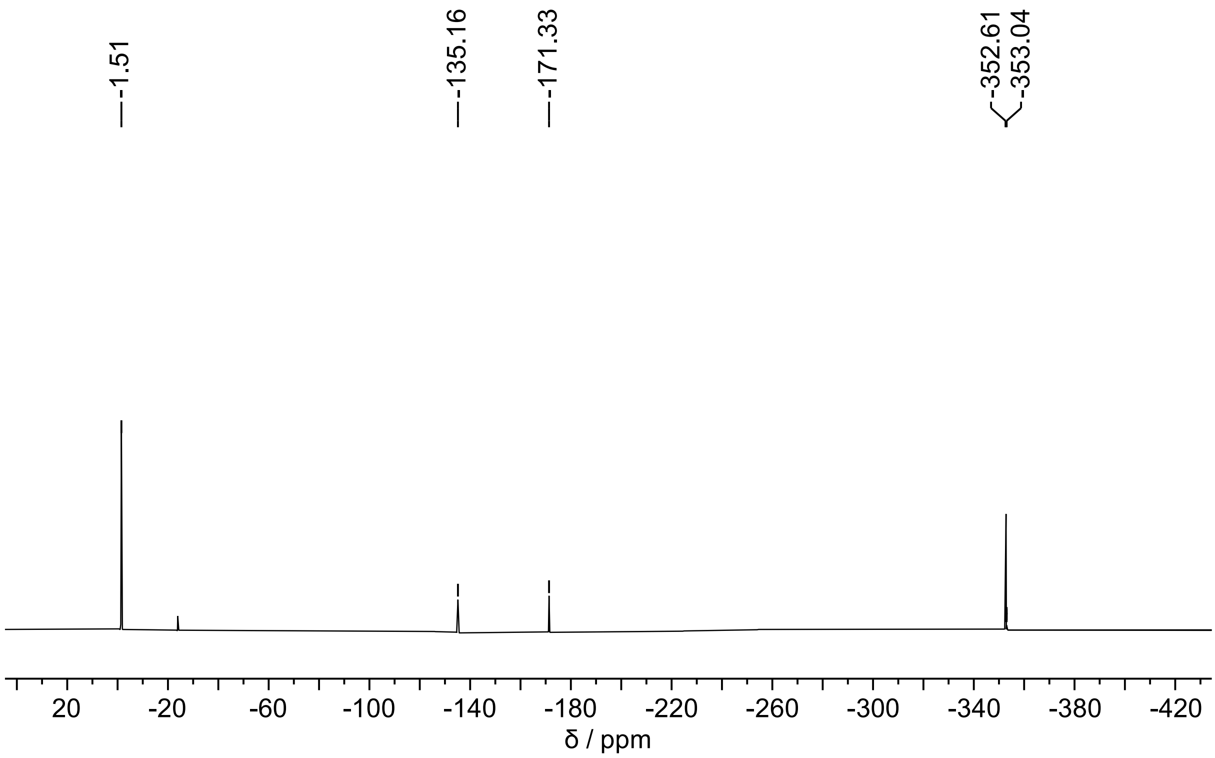

Supplementary Fig. 24: $^{29}Si\{^1H\}$ IG NMR of [K(2.2.2-crypt)][$^{Et}Hyp_3Si_9$] (**2b**) (99.4 MHz, thf-d_8, 300 K).

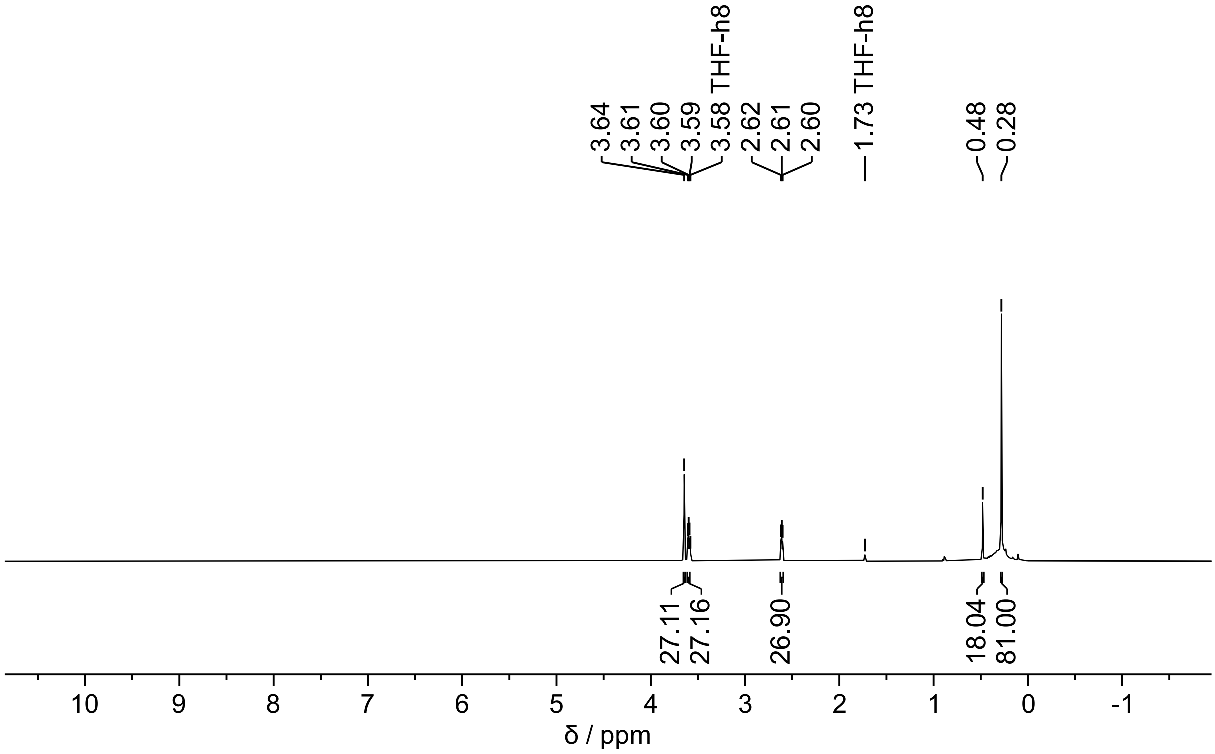

Supplementary Fig. 25: ^1H NMR of [K(2.2.2-crypt)][($^{Me}HypMe_2Si)_3Si_9$] (**2c**) (500 MHz, thf-d_8, 300 K).

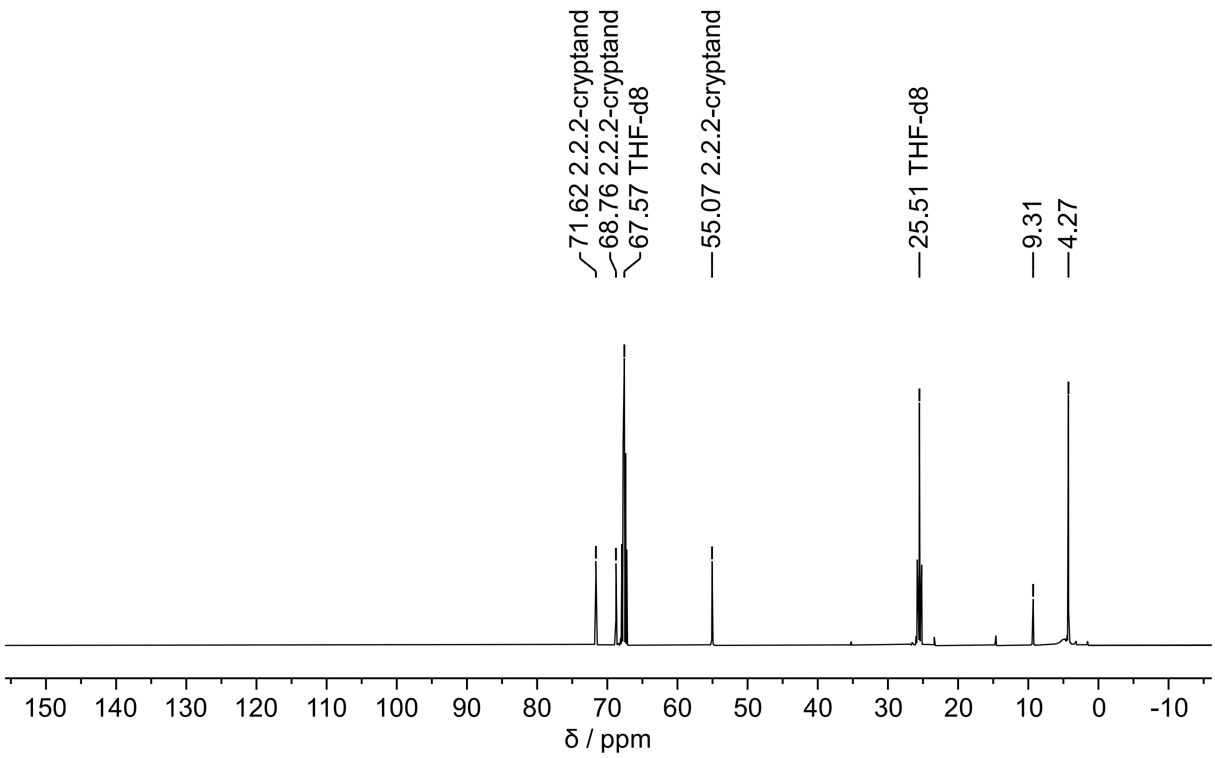

Supplementary Fig. 26: $^{13}C\{^{1}H\}$ NMR of [K(2.2.2-crypt)][($^{Me}HypMe_2Si)_3Si_9$] (**2c**) (126 MHz, thf-d_8, 300 K).

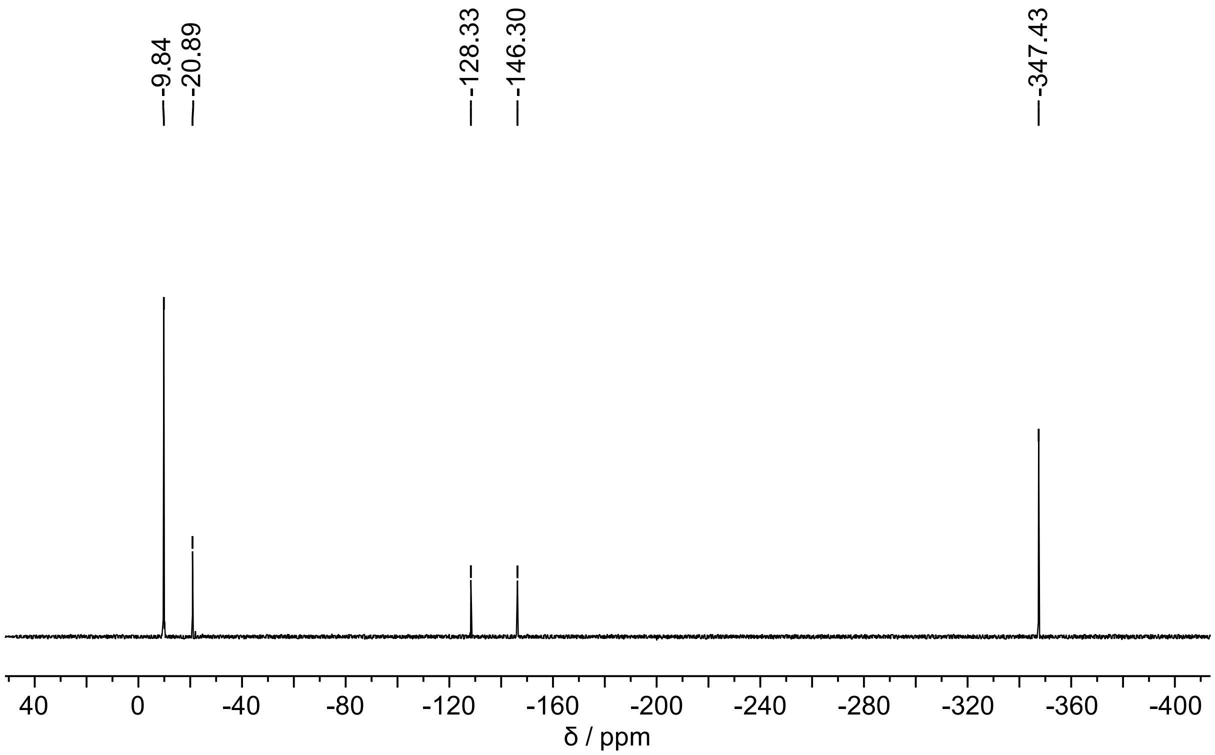

Supplementary Fig. 27: $^{29}Si\{^{1}H\}$ IG NMR of [K(2.2.2-crypt)][($^{Me}HypMe_2Si)_3Si_9$] (**2c**) (99.4 MHz, thf-d_8, 300 K).

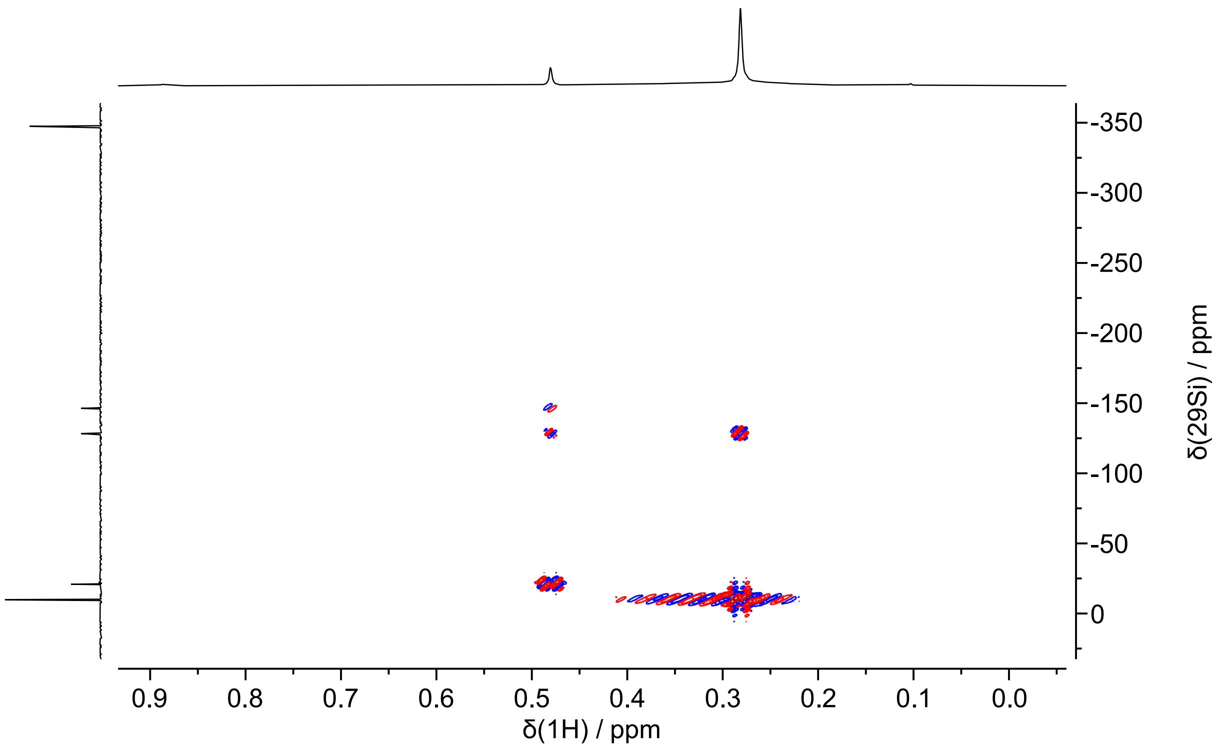

Supplementary Fig. 28: ^{1}H ^{29}Si HMBC of [K(2.2.2-crypt)][($^{Me}HypMe_2Si)_3Si_9$] (**2c**) (500 MHz, thf-d_8, 300 K).

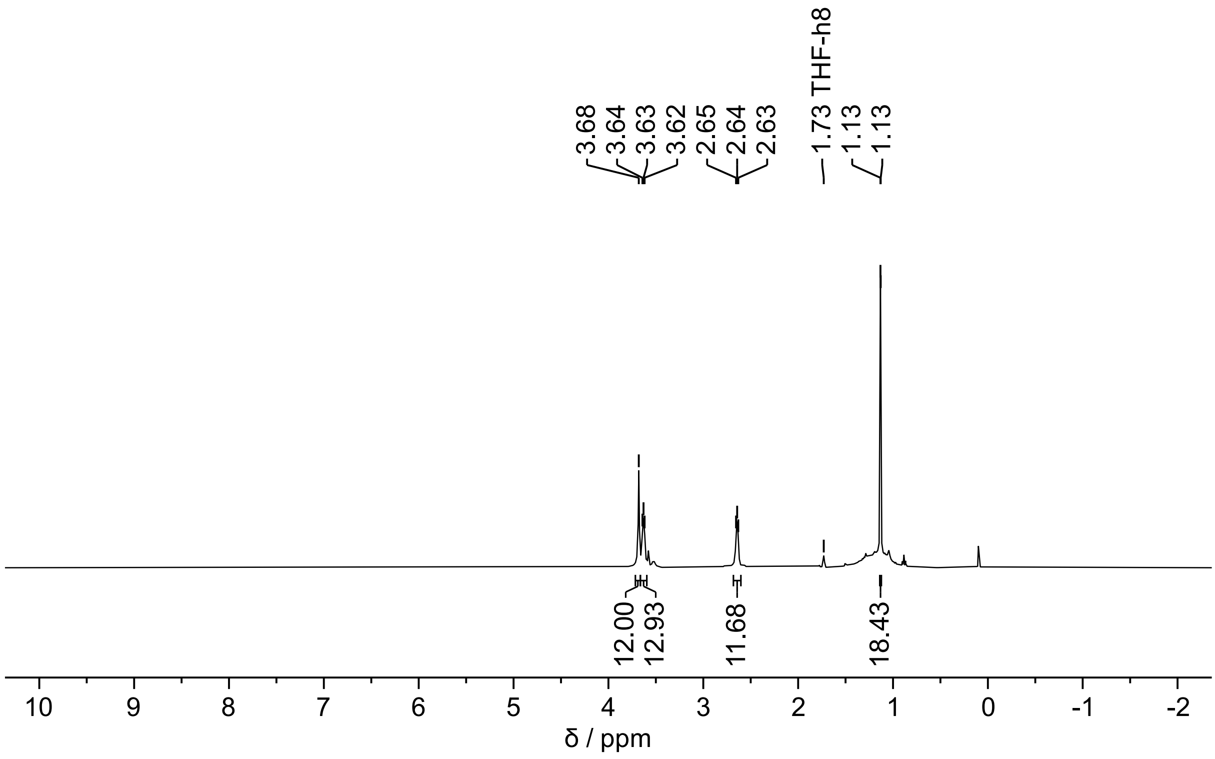

Supplementary Fig. 29: ^{1}H NMR of [K(2.2.2-crypt)][($^{t}Bu_2FSi)_3Si_9$] (**2d**) (500 MHz, thf-d_8, 300 K).

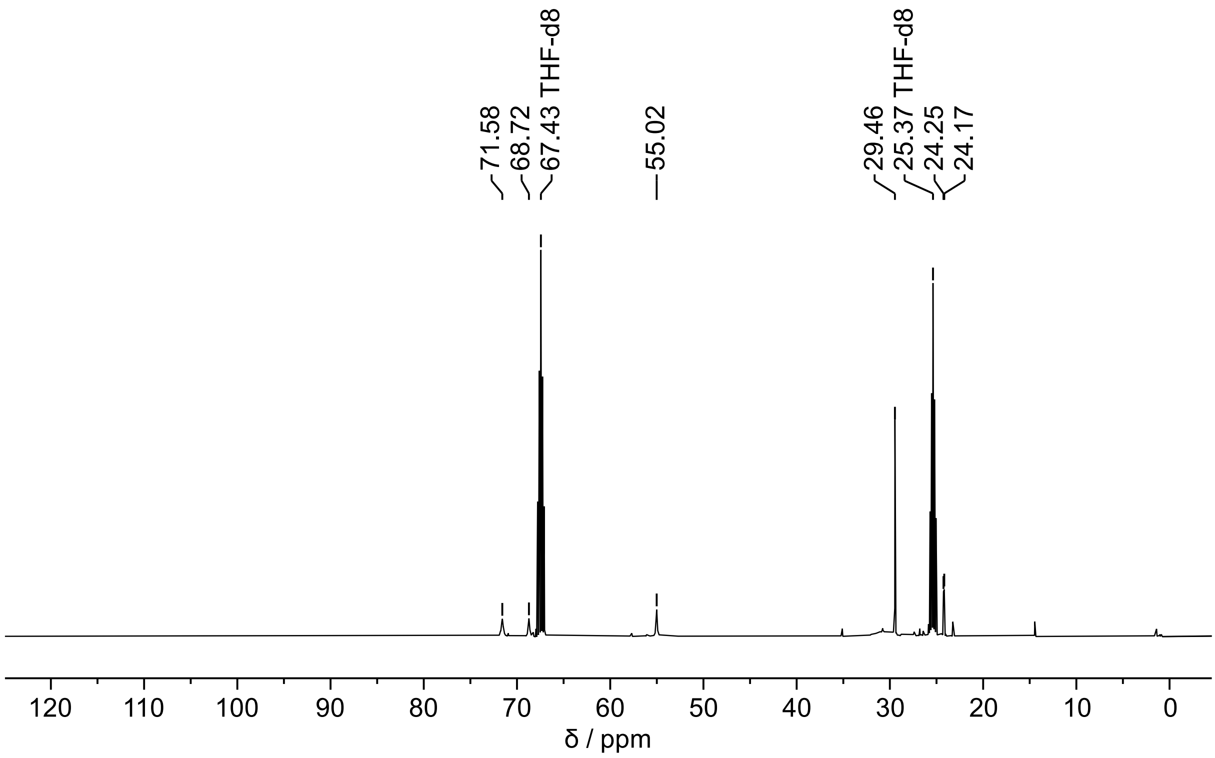

Supplementary Fig. 30: $^{13}C\{^1H\}$ NMR of [K(2.2.2-crypt)][(^{t}Bu$_2$FSi)$_3$Si$_9$] (**2d**) (126 MHz, thf-d_8, 300 K).

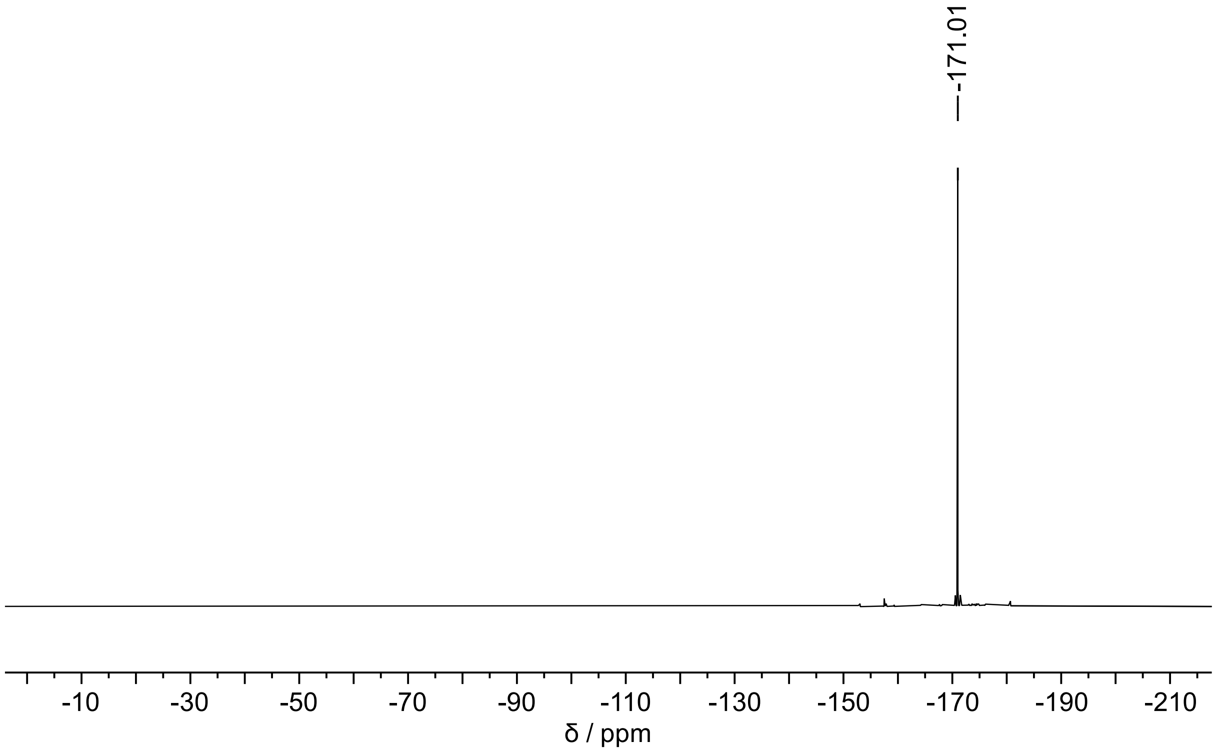

Supplementary Fig. 31: $^{19}F\{^1H\}$ NMR of [K(2.2.2-crypt)][(^{t}Bu$_2$FSi)$_3$Si$_9$] (**2d**) (470 MHz, thf-d_8, 300 K).

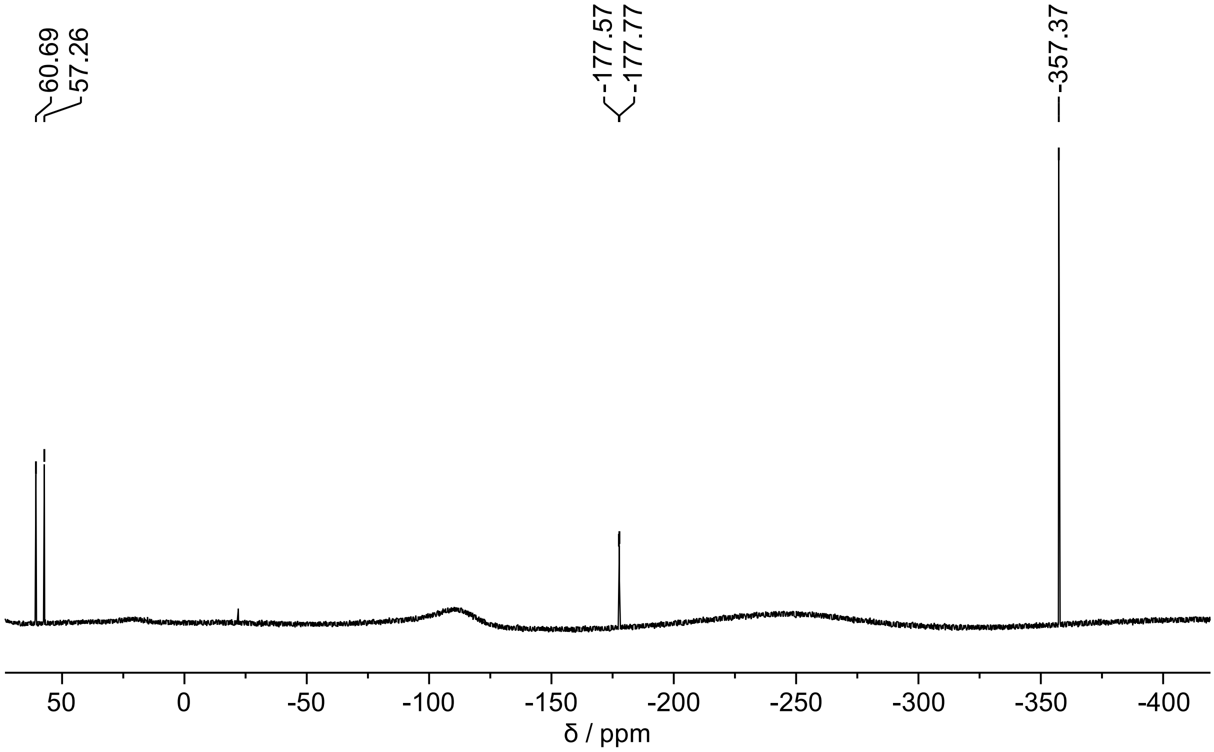

Supplementary Fig. 32: $^{29}Si\{^1H\}$ IG NMR of [K(2.2.2-crypt)][(^{t}Bu$_2$FSi)$_3$Si$_9$] (**2d**) (99.4 MHz, thf-d_8, 300 K).

5. UV-VIS spectra

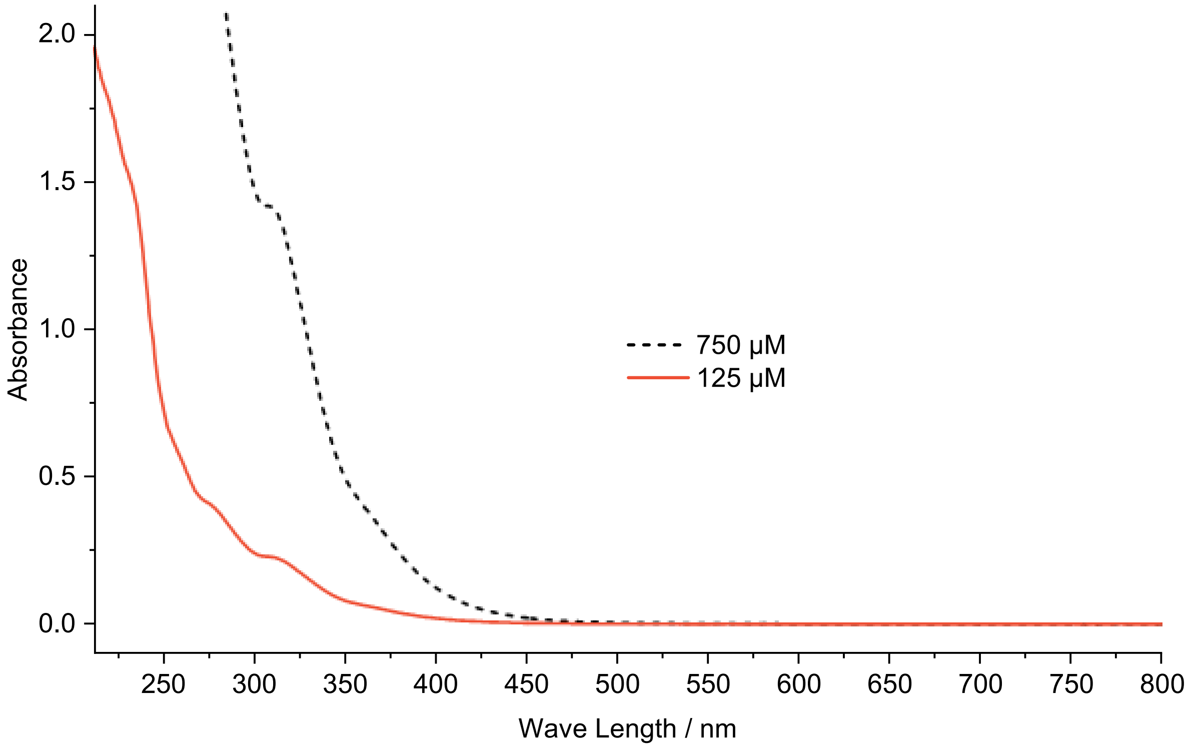

Supplementary Fig. 33: UV-VIS spectrum of [K(2.2.2-crypt)][$^{Me}Hyp_3Si_9$] (**2a**) (300 K, thf).

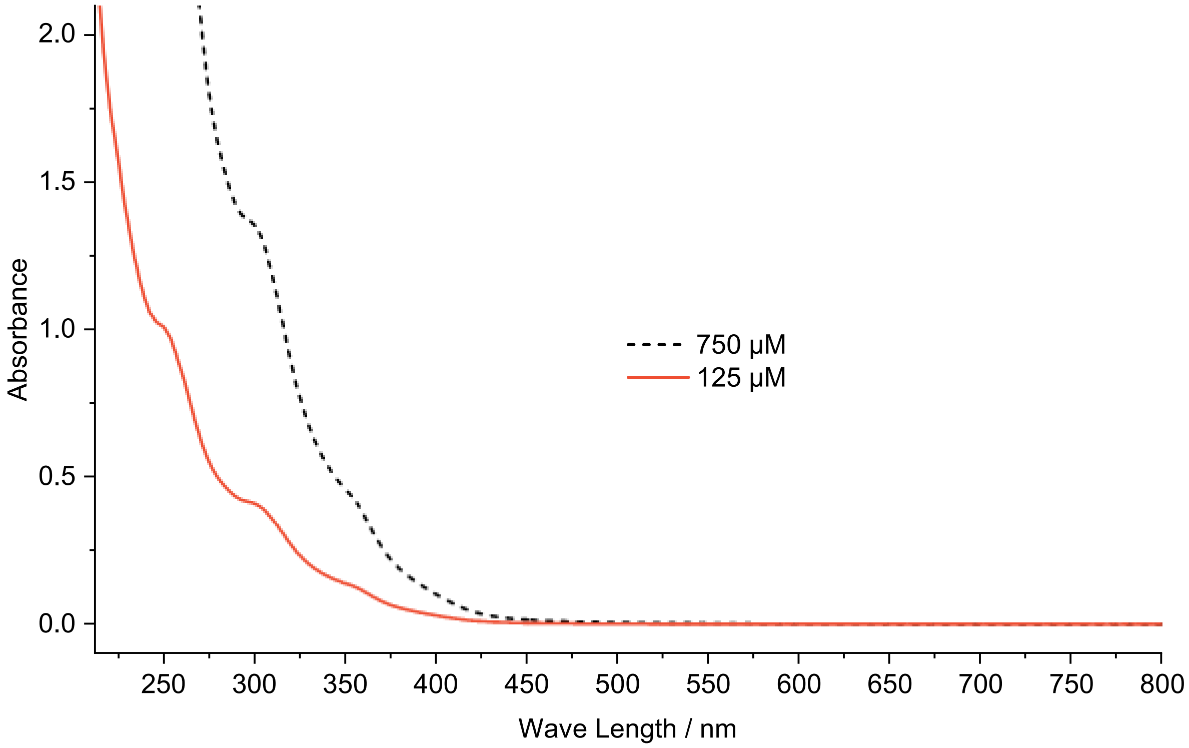

Supplementary Fig. 34: UV-VIS spectrum of [K(2.2.2-crypt)][($^{t}Bu_2FSi)_3Si_9$] (**2d**) (300 K, thf).

6. Review discussion

Reviewer #2 (Remarks to the Author):

The authors have made many of the suggested corrections. Of particular interest are the deuterium labelling studies suggested by reviewer #1. These certainly improve the manuscript. The mass-spec data are still ambiguous, and the elemental analysis data for $K[K(2.2.2\text{-crypt})]_2[Si_9H]$ is, according to the authors, contaminated by 2.2.2-crypt, and thus does not provide conclusive evidence of the compositional purity of the sample.

Answer:

Thank you for giving us once more the opportunity to clarify these important points. The mass spectrometric analysis of the filtrate primarily provides evidence for the integrity of the nine-atomic cluster framework and clearly shows that intact Si_9 clusters are obtained after filtration. It is important to note that we do not claim at any point in our manuscript that the detection of protonated cluster species in the gas phase is taken as proof of a specific protonation state ($[Si_9H]^{3-}$ or $[Si_9H_2]^{2-}$). Proton transfers can always occur during evaporation of the solution in the mass spectrometer. Therefore, this would be an overinterpretation of the mass spectrometric data and is particularly inadmissible. Again, we do not use the mass spectrometric data for the distinction between mono- or bis-protonated clusters, but the data merely confirm the presence of nine-atomic silicon clusters in the filtrate. Only our extensive and unequivocal NMR spectroscopic and SC-XRD studies show that the silicon clusters can be attributed to monoprotonated $[Si_9H]^{3-}$ ions rather than $[Si_9H_2]^{2-}$ ions in the filtrate. Thus, we can on one hand refine the single proton directly at the cluster framework due to the good data quality, and on the other hand determine the charge state of -3 based on the number of $[K(2.2.2\text{-crypt}]^+$ ions in the asymmetric unit. Consequently, the mass spectrometric data are just one part of the overall analysis. Any possible ambiguities of this individual analysis are unequivocally excluded by the entirety of all investigations.

The elemental analysis of the unpurified filtrate shows a remarkably high agreement with $K[K(2.2.2\text{-crypt})]_2[Si_9H]$. We want to emphasise that this is the raw product directly after filtration in liquid ammonia of highly reactive species. Nevertheless, we find the clusters form salts of partially sequestered potassium ions which had been written in a general form as $K[K(2.2.2\text{-crypt})]_2[Si_9H]$. In a non-crystalline material the exact number of crypt molecules can vary. In the present case the elemental analysis would match perfectly with $K_{1-x}[K(2.2.2\text{-crypt})]_{2+x}[Si_9H]$ for x = 0.2.

However, we completely agree with you that the exact composition of the crude product cannot be determined based on elemental analysis alone, and it cannot be considered as a analytically pure product. Instead, we believe that the composition of the dried residue is approximately $K_{1-x}[K(2.2.2\text{-crypt})]_{2+x}[Si_9H]$ and may be subject to slight variations in cryptand content. Only the addition of more cryptand allows for the isolation of a completely pure substance, namely $[K(2.2.2\text{-crypt})]_3[Si_9H]$ 8.5 NH_3. We like to point out however, that the goal of our work is to provide a simple and reliable access to a starting material containing solely silicon nine-atoms clusters, which was impossible until now. We believe it is important to consider the filtrate as an intermediate step from the solid phase to silylated, pure cluster species. Given the reproducibility of our syntheses, the high purities and yields of our silylated target products (**2**), and the remarkably high correspondence of the elemental analysis of the filtrate, we are convinced that $K_{1-x}[K(2.2.2\text{-crypt})]_{2+x}$[Si9H] is a valid description of the raw material. The fact that we cannot crystallise the filtrate without adding an additional equivalent of 2.2.2-cryptand to form $[K(2.2.2\text{-crypt})]_3[Si_9H]$ (x = 1) further suggests that the filtrate can be described as $K_{1-x}[K(2.2.2\text{-crypt})]_{2+x}$[Si9H]. We added in the revised version: “Although we can clearly demonstrate that four- and nine-atom clusters can be separated by this procedure, the exact chemical composition of the dried filtrate cannot be conclusively determined. Elemental analysis of the solid suggests a composition corresponding to $K_{1-x}[K(2.2.2\text{-crypt})]_{2+x}[Si_9]$ (x = 0.2), indicating a cluster with a reduced charge of 3–. Clusters with this charge have been observed for both paramagnetic $[Si_9]^{3-}$ [68] and protonated $[Si_9H]^{3-}$ [71] in ammonia. We would like to point out that the solid is not a pure intermediate but rather a crude product. The exact 2.2.2-crypt content in this intermediate after filtration cannot be conclusively determined and may vary around an ideal composition of $K_1[K(2.2.2\text{-crypt})]_2[Si_9]$. Therefore, we can only postulate a composition of $K_{1-x}[K(2.2.2\text{-crypt})]_{2+x}[Si_9]$. Crystallisation of the crude product from DMF, DMP, HMPA, and ammonia was not possible.”

7. References

1. Schiegerl, L. J., Karttunen, A. J., Klein, W. & Fässler, T. F. Silicon clusters with six and seven unsubstituted vertices via a two-step reaction from elemental silicon. *Chem. Sci.* **10**, 9130-9139, (2019).
2. Goicoechea, J. M. & Sevov, S. C. Naked deltahedral silicon clusters in solution: synthesis and characterization of Si_9^{3-} and Si_5^{2-}. *J. Am. Chem. Soc.* **126**, 6860-6861, (2004).
3. Kysliak, O. & Schnepf, A. $\{Ge_9[Si(SiMe_3)_3]_2\}^{2-}$: a starting point for mixed substituted metalloid germanium clusters. *Dalton Trans.* **45**, 2404-2408, (2016).
4. Li, F. & Sevov, S. C. Rational synthesis of $[Ge_9\{Si(SiMe_3)_3\}_3]^-$ from its parent zintl ion Ge_9^{4-}. *Inorg. Chem.* **51**, 2706-2708, (2012).
